# Cancer selective cell death induction by a bivalent CK2 inhibitor targeting the ATP site and the allosteric αD pocket

**DOI:** 10.1016/j.isci.2024.108903

**Published:** 2024-01-12

**Authors:** Alexandre Bancet, Rita Frem, Florian Jeanneret, Angélique Mularoni, Pauline Bazelle, Caroline Roelants, Jean-Guy Delcros, Jean-François Guichou, Catherine Pillet, Isabelle Coste, Toufic Renno, Christophe Battail, Claude Cochet, Thierry Lomberget, Odile Filhol, Isabelle Krimm

**Affiliations:** 1 University Lyon, Université Claude Bernard Lyon 1, INSERM 1052, CNRS 5286, Centre Léon Bérard, Centre de recherche en cancérologie de Lyon, Institut Convergence Plascan, Team « Small Molecules for Biological Targets », 69373 Lyon, France; 2 University Lyon, Université Claude Bernard Lyon 1, INSERM 1052, CNRS 5286, Centre Léon Bérard, Centre de recherche en cancérologie de Lyon, Institut Convergence Plascan, Team « Targeting Non-canonical Protein Functions in Cancer », 69373 Lyon, France; 3 Université Grenoble Alpes, IRIG, Laboratoire Biosciences et Bioingénierie pour la Santé, UA 13 INSERM-CEA-UGA, 38000 Grenoble, France; 4 University Grenoble Alpes, INSERM 1292, CEA, UMR Biosanté, 38000 Grenoble, France; 5 Centre de Biologie Structurale, CNRS, INSERM, University Montpellier, 34090 Montpellier, France; 6 University Lyon, Université Claude Bernard Lyon 1, CNRS UMR 5246, Institut de Chimie et Biochimie Moléculaires et Supramoléculaires (ICBMS), COSSBA Team, Faculté de Pharmacie-ISPB, 8 Avenue Rockefeller, 69373 Lyon Cedex 08, France; 7 Kairos Discovery SAS, 36 Rue Jeanne d’Arc, 69003 Lyon, France

**Keywords:** Molecular biology, Cell biology, Structural biology, Cancer

## Abstract

Although the involvement of protein kinase CK2 in cancer is well-documented, there is a need for selective CK2 inhibitors suitable for investigating CK2 specific roles in cancer-related biological pathways and further exploring its therapeutic potential. Here, we report the discovery of AB668, an outstanding selective inhibitor that binds CK2 through a bivalent mode, interacting both at the ATP site and an allosteric αD pocket unique to CK2. Using caspase activation assay, live-cell imaging, and transcriptomic analysis, we have compared the effects of this bivalent inhibitor to representative ATP-competitive inhibitors, CX-4945, and SGC-CK2-1. Our results show that in contrast to CX-4945 or SGC-CK2-1, AB668, by targeting the CK2 αD pocket, has a distinct mechanism of action regarding its anti-cancer activity, inducing apoptotic cell death in several cancer cell lines and stimulating distinct biological pathways in renal cell carcinoma.

## Highlights

• AB668: a selective bivalent CK2 inhibitor targeting the ATP site and the αD pocket
• Treatment of cancer cells with AB668 induces cell death through caspase3 activation
• AB668 stimulates different biological pathways compared to CK2 ATP-site inhibitors

## Graphical abstract

**Figure:**
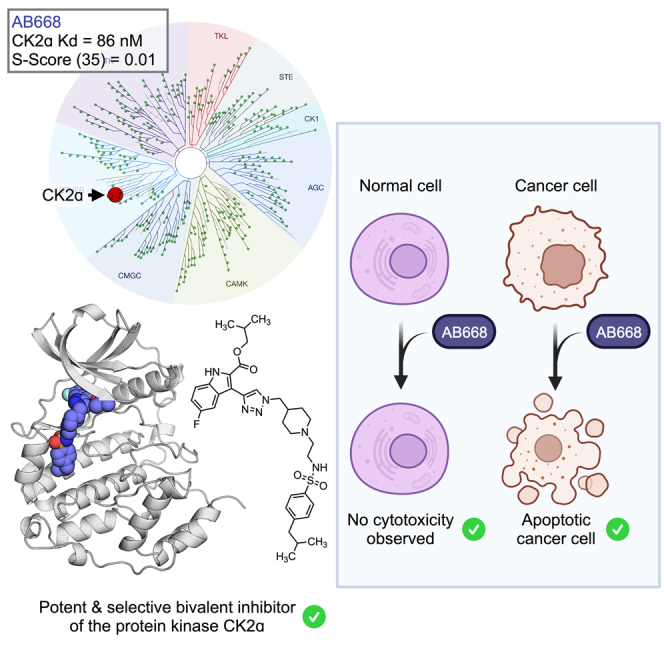

## Introduction

CK2 is a constitutively active protein kinase ubiquitously expressed in eukaryotes and particularly well conserved among species. CK2 phosphorylates serine or threonine residues within an acidic context (S/TXXD/E/pS/pT/pY) and is responsible for the generation of a large proportion of the human phosphoproteome.^1^ One of the distinctive features of CK2 is to exist in cells both as a catalytic subunit CK2α and as a holoenzyme CK2α_2_β_2_, which consists of two catalytic subunits CK2α that interact with a dimer of two regulatory subunits CK2β.^2^ Although the so-called regulatory CK2β subunits are not essential per se for CK2α kinase activity as the catalytic subunit is constitutively active,^3^ several reports showed that CK2β influences significantly CK2 substrate preference as well as CK2α localization.^4^^,^^5^ Up to now, several hundreds of proteins have been shown to be CK2 substrates in cells.^6^^,^^7^^,^^8^ CK2 is involved in important physiological functions, such as embryonic development, differentiation, immunity, cell survival, epithelial homeostasis, and circadian rhythms.^9^ CK2 is also implicated in numerous human diseases such as cancer, neurodegenerative diseases, viral and parasite infections, cystic fibrosis, psychiatric disorders, diabetes, inflammatory, and cardiovascular diseases.^10^ In cancer, CK2 promotes cell proliferation and survival.^11^^,^^12^ CK2 might also be involved in immune cell development and function in cancer,^13^ in cancer metabolism,^14^ as well as in antitumor drug resistance.^15^ For example, proliferation, migration, invasion, and survival of cholangiocarcinoma cells exposed to cytostatic drugs are markedly reduced when cells are depleted in CK2α subunit.^16^ Globally, CK2 is overexpressed in a very large number of human tumors (e.g., breast, ovarian, prostate, lung, colon, kidney, skin, and pancreatic cancers) and its over-expression correlates with poor prognosis.^17^^,^^18^^,^^19^ The higher sensitivity of cancer cells to CK2 inhibition, as compared to their healthy counterparts, led to the hypothesis of a “non-oncogenic” CK2 addiction of cancer cells.^20^^,^^21^ Compiling evidence from the literature suggests that CK2 modulates all hallmarks of cancer.^22^ Consequently, CK2 is considered as a “master regulator” and a promising therapeutic target to treat different human tumors.^23^

Many CK2 inhibitors that target the CK2 catalytic site have been proposed.^24^ The most advanced molecule silmitasertib (CX-4945) inhibits CK2α catalytic activity with a K_i_ of 0.38 nM,^25^ and the compound has entered several clinical trials. Notably, silmitasertib received an orphan drug designation for the treatment of cholangiocarcinoma in 2016, medulloblastoma in 2020, recurrent Sonic Hedgehog-driven medulloblastoma in 2021, and biliary tract cancer in 2022.^26^ Although described as highly selective, CX-4945 inhibits several kinases with nanomolar IC_50_ values (CLK1, CLK2, CLK3, DYRK1A, DYRK1B, DAPK3, HIPK3 …).^25^ For example, CX-4945 was reported to regulate splicing in mammalian cells in a CK2-independent manner through the inhibition of Clk1, Clk2, and Clk3.^27^ Therefore, despite its therapeutic efficacy, CX-4945 cannot be used to probe the cellular functions of CK2. Recently, an ATP-competitive CK2 inhibitor, SGC-CK2-1, was reported.^28^ SGC-CK2-1 (IC_50_ 2.3 nM on CK2α) is much more selective than CX-4945 and was consequently described as a genuine chemical probe to assess the consequences of the pharmacological inhibition of CK2 kinase activity.^28^ When tested on a panel of 140 cancer cell lines, SGC-CK2-1 reduced cell growth in blood, head/neck, brain, breast, skin, stomach, and duodenum cell lines with a micromolar range efficacy. This poor antiproliferative effect led the authors to question the inhibition of CK2 as a strategy for cancer therapy.^28^^,^^29^

To further decipher the role of CK2 in cancer biology, we considered the opportunity of targeting allosteric sites of the protein. Allosteric compounds have the advantage to bind to less conserved pockets of the kinase. Conceptually, such compounds would be more selective chemical tools. Also, as therapeutics, they would generate less side effects due to off-target kinase inhibition.^30^ Regarding CK2, the CK2α/CK2β interface and the so-called αD pocket have both been targeted by small-molecule inhibitors (Figure S1).^31^ We previously reported a small molecule that binds at the CK2α/CK2β interface and disrupts the holoenzyme in cells, inhibiting cell growth and migration.^32^ However, the affinity of the inhibitor was rather weak (K_D_ 30 μM) for further characterization. Bivalent CK2 inhibitors targeting simultaneously the ATP-binding site and the αD pocket have also been reported.^33^^,^^34^^,^^35^ The bivalent inhibitor CAM4066 (K_D_ of 0.32 μM and IC_50_ of 0.37 μM) reduced cell viability in HCT116, Jurkat, and A549 cells with GI _50_ of 9.6 and 20 μM, respectively.^33^ The bivalent inhibitor KN2 (K_i_ 6 nM for CK2 holoenzyme) proved to be cytotoxic in HeLa cells (6 μM) but also displayed cytotoxicity in nontumor HEK293 cell line (16 μM).^35^ Importantly, both bivalent inhibitors showed high selectivity when tested against a panel of 52 and 83 diverse kinases for CAM4066 (2 μM) and KN2 (3 μM), respectively.

Here, we report a highly selective CK2 bivalent inhibitor, AB668, which binds simultaneously the ATP site and the αD pocket of CK2α. Our aim was to propose a chemical tool to explore CK2 function in cancer cells and compare pure ATP-based CK2 inhibition with bivalent-based CK2 inhibition. The bivalent inhibitor AB668, which inhibits the activity of the CK2 holoenzyme with a K_i_ of 41 nM, displays an outstanding selectivity measured against a panel of 468 kinases. AB668 therefore represents a valuable chemical tool to explore CK2 mechanisms in cancer and to decipher the specific function of this particular pocket of the kinase. Here, we mainly compared the impact of AB668 and the two ATP-competitive CK2 inhibitors CX-4945 and SGC-CK2-1 on 786-O renal carcinoma cells and human melanoma cells, using live cell imaging and transcriptomic analysis. Our results highlight that, by targeting both the ATP site and the αD pocket, AB668 discloses a distinct mode of action in cancer cells compared to canonical ATP-competitive inhibitors, suggesting that targeting the αD pocket of CK2 may represent a valuable pharmacological tool and therapeutic strategy in cancer.

## Results

### AB668 interacts with the ATP site and αD-pocket of CK2

**Figure 1:**
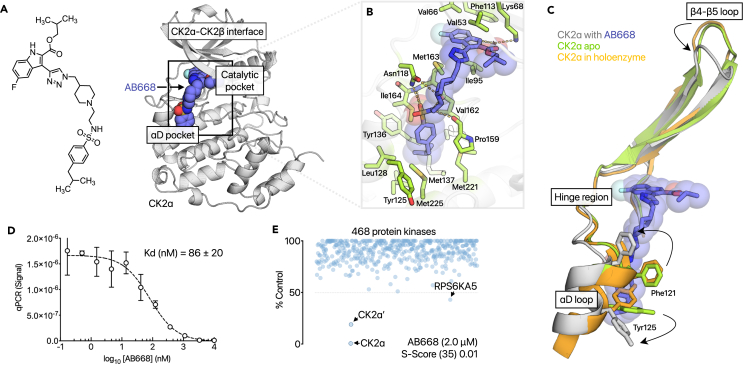
Binding mode, affinity for CK2α and kinase selectivity profile of AB668 (A) Chemical structure of AB668 and CK2α/AB668 complex crystal 3D structure (PDB: 8C5Q) showing the binding site of AB668: the inhibitor binds both the ATP pocket and the αD pocket of CK2α. (B) Stick representation of AB668 bound to CK2α: side chains in interaction with the inhibitor are shown, and hydrogen bonds are displayed. (C) Conformational rearrangement of CK2α upon AB668 binding: the helix is shifted to allow the binding of AB668 in the αD pocket. The structure of CK2α bound to AB668 is superimposed to the structure of the holoenzyme (PDB: 1JWH), and to the apo structure of CK2α (PDB: 3QAO). (D) Binding affinity of AB668 as determined by the KINOME*scan* profiling service (Eurofins). Data are mean of 2 independant experiments. (E) Selectivity profile of AB668, profiled against 468 kinases, using the screening platform from Eurofins DiscoverX. AB668 concentration was 2 μM (25 times its K_d_ value). See also Figures S1–S8 and Table S1.

During our efforts to optimize a CK2α/CK2β interface inhibitor that we previously reported,^32^ (Figure S1), one of the chemical series containing a triazole group led to the discovery of AB668, a compound that simultaneously binds at the ATP site and the αD allosteric pocket of CK2 (Figures 1A and S2–S5). A thermal shift assay experiment was used to confirm direct binding of AB668 with CK2α. CK2α alone displayed a Tm of 43.6 ± 0.4°C. At saturating inhibitor concentration, AB668 induced a significant increase of the Tm value (ΔTm = 5.2 ± 0.4°C). By comparison, the ATP-competitive inhibitors CX-4945 and SGC-CK2-1 induced a substantial increase of the Tm value (ΔTm = 13.8 ± 0.4°C, ΔTm = 11.0 ± 0.3°C, respectively) (Figures S6A and S6B). The lower magnitude of the change in Tm induced by AB668 may be related to its different binding site and to its lower affinity, as described in the following section. The 3D X-ray structure of AB668 bound to CK2α was resolved using crystallization conditions reported for the X-ray structure of the CK2α-SGC-CK2-1 complex.^28^ The indole moiety of the bivalent inhibitor AB668 binds in the ATP site and interacts with the side chain of Lys68 through a hydrogen bond mediated by a water molecule, and the carbonyl group directly interacts with Lys68 through a weak hydrogen bond (Figure 1B). The aromatic moiety of indole is sandwiched between Val53, Val66, Ile95, Phe113, Met163, and Ile174. On the other side of the bivalent inhibitor, the substituted phenyl binds in the hydrophobic αD-pocket of CK2 (Tyr125, Leu128, Ile133, Met137, Tyr136, Ile140, Pro159, Val162, Ile164, Met221, and Met225). Regarding the linker between the indole moiety and the substituted phenyl, the sulfonamide group interacts with the peptide chain of Ile164, while the nitrogen of piperidine interacts with Asn118 side chain. As illustrated in Figure 1C, the αD-helix position is shifted together with the side chains of residues Phe121 and Tyr125 upon AB668 binding and remains flexible in the crystal, with large B-factors values observed from residues Asn118 to Thr127. This conformational changed is similar to the one previously observed on CK2α and CK2α′ when bound to CAM4086 and KN2.^33^^,^^34^^,^^35^ Modification of the conformation of the β4-β5 loop is observed in one monomer but not in the second one, suggesting that the loop conformation is highly dynamic, which is corroborated by its large B-factors.

### AB668 inhibits CK2 activity with an outstanding selectivity

AB668 inhibited the CK2 holoenzyme with a K_i_ value of 41 nM (IC_50_ 65 nM), as determined using a canonical radiometric assay with a CK2β-dependent peptide substrate (Figure S7A). By comparison, CX-4945 and SGC-CK2-1 inhibited the isolated CK2α subunit with K_i_ of 6.2 nM and 4.5 nM, respectively, in our experimental conditions (Figure S6C). We also showed that AB668 inhibits the phosphorylation of a CK2 protein substrate. The phosphorylation of SIX1, a transcription factor that is specifically phosphorylated by the CK2 holoenzyme,^36^ was inhibited by about 90% in the presence of 0.5 μM AB668 (Figure S7B).

The affinity of AB668 for CK2α was 86 ± 20 nM, as determined by the KINOME*scan* profiling assay (Eurofins) (Figure 1D). This assay is an active site-directed competition binding assay that does not require ATP and reports thermodynamic interaction affinities.

The selectivity of AB668 was profiled against 468 kinases, using an active site-directed competition binding assay with the screening platform KINOME*scan* from Eurofins DiscoverX. For this, AB668 was tested at a concentration of 2 μM (25 times its K_D_ value). In this assay, an ATP-site kinase ligand is immobilized to perform an active site-directed competition binding assay. The DNA-tagged kinases are captured in the absence or presence of AB668. Competition is measured using a qPCR method that detects the associated DNA label. In addition, dissociation constants were measured using the same assay for the kinases MARK3 and PIKFYVE as the screening assay indicated a strong inhibition comparable to CK2α and CK2α’. K_D_ values larger than 10 μM were reported for MARK3 and PIKFYVE.

Therefore, as shown in Figure 1E, besides CK2α and CK2α′, only one kinase (RPS6KA5) displayed a percentage inhibition larger than 50%. A value of 0.01 was obtained for the selectivity score (S_10_[2 μM]),^37^ showing that, by targeting the ligandable αD pocket, AB668 displays an outstanding selectivity against a large kinase panel. Although assessed on a more limited set of kinases, a very high selectivity was also previously reported for bivalent inhibitors CAM4066 (2 μM) and KN2 (3 μM), both targeting the αD-pocket.^33^^,^^34^^,^^35^

KINOME*scan* profiling indicated that CK2α′ was slightly less sensitive to AB668 than CK2α (81% and 99.4% inhibition in the presence of 2 μM AB668, respectively (Figure 1E). However, using a radiometric assay, we found a very similar efficacy of AB668 on the catalytic activity of both isoforms (Figure S8). These results are consistent with the high sequence identity between the two isoforms in the αD pocket. The residues Tyr125, Leu128, Ile133, Tyr136, Met137, Pro159, Val162, Ile164, Met221, and Met225 are conserved while Ile140 in CK2α is replaced by Leu 141 in CK2α'. In addition, in the ATP binding site, residues His115 and Val116 in CK2α are replaced by Tyr 116 and Ile117 in CK2α'. Of note, the bivalent inhibitor KN2 was shown to inhibit CK2α_2_β_2_ and CK2α′_2_β_2_ with similar K_i_ values (6.1 ± 2.0 nM and 4.0 ± 1.4 nM, respectively).^35^

### AB668 induces apoptotic cell death in cancer cells in contrast to SGC-CK2-1

**Figure 2:**
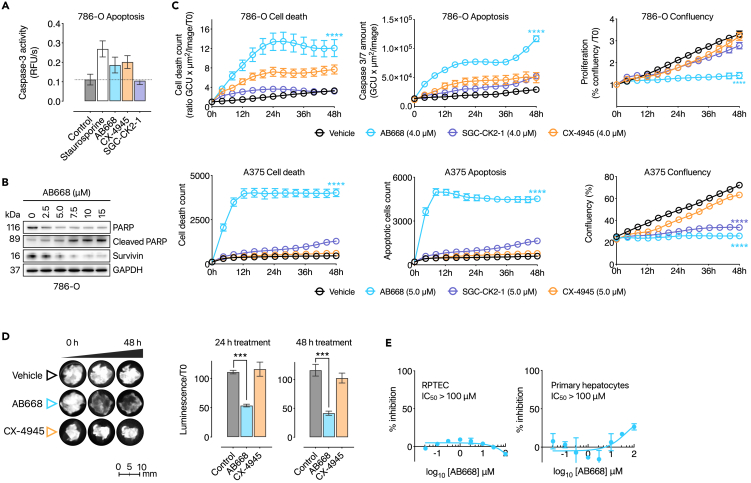
Cellular activity of AB668 and comparison to CX-4945 and SGC-CK2-1 (A) Quantitative fluorometric assay showing caspase-3 activation in 786-O cells treated with staurosporine, AB668, SGC-CK2-1, or CX-4945. Assays were performed after 72 h treatment with the compounds at 20 μM. Data are the mean of 3 independant experiments ± SEM. (B) Western blot experiments on 786-O cells treated with AB668 (2.5, 5, 7.5, 10, and 15 μM) for 48 h showing the cleavage of PARP as well as the expression level of surviving. (C) Live cell imaging showing proliferation arrest, cell death and apoptosis in 786-O and A375 cells treated with AB668, CX-4945 or SGC-CK2-1 (4 μM) for 48h. Statistical analysis was made using Kruskal-Wallis one-way ANOVA n: 12 images per condition over time, ∗p > 0.05; ∗∗∗p > 0.001 and ∗∗∗∗p > 0.0001. (D) Effect of AB668 and CX-4945 on *ex vivo* culture of intact tumor slices of clear cell renal carcinoma. Tumors were extracted from renal carcinoma xenografted mice that were directly processed into 300 μm slices and treated for 48 h as described in STAR Methods. Cell viability was evaluated by luciferin measurement of treated tumor-slice cultures (right panel) as described in STAR Methods. Statistical analysis was made using Kruskal-Wallis one-way ANOVA n: 3 slices per treatment, ∗∗∗p < 0.001. (E) Cell viability of RPTEC (renal proximal tubule epithelial cells) and primary hepatocytes treated with increasing concentrations of AB668. Data shown are the mean of 3 independent experiments ±SEM. Statistical analysis was made using Student’s *t* test, ∗p > 0.05; ∗∗∗p > 0.001 and ∗∗∗∗p > 0.0001. See also Figures S9–S11.

We next evaluated whether AB668 was capable of engaging and inhibiting CK2 in living cells. As we previously reported that the CK2 subunits are overexpressed at the protein level in renal carcinoma compared to normal renal tissues,^19^ we tested the effects of AB668, CX-4945, and SGC-CK2-1 in 786-O renal carcinoma cells. Like staurosporine, AB668 and CX-4945 induced caspase-3 activation in 786-O cells, as shown after 72 h treatment using a quantitative fluorometric assay (Figure 2A). In contrast, and as previously reported,^28^ SGC-CK2-1 did not activate caspase-3 (Figure 2A). Caspase-3 activation induced by AB668 was confirmed by western blot experiments (Figure 2B), showing the cleavage of PARP (poly (ADP-ribose) polymerase), a known target of caspase-3.^38^^,^^39^ Similarly, AB668 reduced the expression of survivin, a member of the inhibitor of apoptosis arotein family that inhibits caspases and blocks cell death (Figure 2B).^40^ These observations encouraged us to further characterize the potential functional impact of AB668 and the ATP-competitive inhibitors in living cancer cells.

For this, Incucyte live-cell analysis was used to evaluate the effect of the various CK2 inhibitors in 786-O cells. Dose dependent experiments were performed for the three inhibitors, indicating that at a low concentration (<5μM), AB668 was more efficient than CX-4945 and SGC-CK2-1 to induce renal cancer cell death (Figure S9). We choose 4 μM concentration to compare the effect of the three inhibitors on proliferation, cell death, and apoptosis in 786-O cells. We also performed the experiments on a melanoma cell line (A375), for which some information is available regarding the effect of CK2 inhibition.^41^^,^^42^ As illustrated in Figure 2C, AB668 induced significant cell proliferation arrest associated with cell death and apoptosis in both cancer cell lines. By comparison, CX-4945 had only a moderate effect on 786-O cell growth and was a poor apoptosis inducer. In contrast, A375 cells were almost completely insensitive to CX-4945 and SGC-CK2-1 as these inhibitors had no impact on the survival of both cell lines (Figure 2C). CK2 has been shown to mediate anti-apoptotic pathway by protecting substrates from caspase-3-mediated proteolysis,^38^^,^^43^ and CX-4945 was previously reported to induce apoptotic cell death in cancer cells lines such as PC3 prostatic adenocarcinoma,^25^ B-ALL, T-ALL,^44^^,^^45^ H1299, Calu-1, and H358.^46^ As both SGC-CK2-1 and CX-4945 target the ATP-site of CK2, it is surprising that the cell treatment with SGC-CK2-1, even at high concentration, does not induce caspase-3-mediated apoptosis (Figure 2A). It is conceivable that caspase-3 activation by CX-4945 is directly or indirectly mediated by its interaction with off-targets. Regarding AB668, the activation of caspase-3 might be related to the disruption of certain cellular pathways caused by the conformational change of the CK2 αD helix when AB668 interacts with CK2α.

We then evaluated the efficacy of AB668 in *ex vivo* renal carcinoma cultures that we previously used to study individual responses to targeted therapies.^47^^,^^48^ As shown in Figure 2D, 10 μM CX-4945 had no effect on tumor slices of renal carcinoma, while 5 μM AB668 significantly reduced cell viability after 24 h of treatment, showing its higher efficacy in this drug sensitivity prediction model. This study suggests that AB668-mediated CK2 inhibition could be a viable therapeutic strategy in renal carcinoma. Importantly, no cytotoxicity of AB668, even at high concentrations, was observed in normal human cell lines such as RPTEC (renal proximal tubule epithelial cells), HEK293 (human embryonic kidney cells), primary hepatocytes, and MCF10A (human breast epithelial cells) (Figures 2E, S10, and S11). Finally, we analyzed the effect of AB668 on five other cancer cell lines (Figure S11), showing that despite the exception of U-373 MG glioblastoma cells, AB668 exhibits a broad effect on cell death in various cancer cells.

### Persistence of cellular effects of CK2 inhibitors

To evaluate the potential reversibility of CK2 inhibition by the 3 different CK2 inhibitors, 786-O cells were treated with 5 μM of each inhibitor for 12 h. Then, cells were washed with PBS and immediately lysed (time 0) or further cultured for 8 h without inhibitor in the medium (time 8). Lysates from treated cells were then analyzed for CK2 activity by radiometric kinase assay (Figure S12A), analysis of radioactively phosphorylated proteins (Figure S12B) or western blot analysis using either an antibody against CK2 substrates (Figure S12C) or the P-AKT(S129) antibody (Figure S12D). These results demonstrate that 8 h after inhibitor removal, the inhibition promoted by AB668 and SGC-CK2-1 persisted whereas, in the case of CX-4945, CK2 activity was restored to its initial level. This is consistent with a study comparing the persistence of the cellular effects promoted by two cell-permeable CK2 inhibitors including CX-4945.^49^

### CK2 downstream cellular events in response to AB668, SGC-CK2-1, and CX-4945

**Figure 3:**
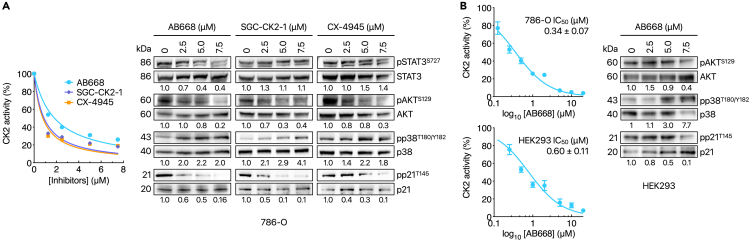
Target engagement of AB668, SGC-CK2-1, and CX-4945 in 786-O cells and HEK293 cells (A) 786-O cells were treated with increasing concentrations of the indicated inhibitors and CK2 activity was measured in cell extracts (left panel). Western blot analysis of the corresponding cell extracts. Phosphorylated STAT3 (S727), p38 MAPK (T180/Y182), and AKT (S129) and p21 (T145) were analyzed after 48 h of treatment (right panel). Data are the mean of 3 independant experiments ± SEM. (B) 786-O and HEK293 cells were treated with increasing concentrations of AB668 and CK2 activity was measured in cell extracts. Inhibition constants IC_50_ are 0.34 ± 0.07 μM for 786-O cells and 0.60 ± 0.11 μM for HEK293 cells (left panel). Phosphorylated AKT (S129), p38 MAPK (T180/Y182), and p21 (T145) were analyzed after 48 h of AB668 treatment of HEK293 cells. Data shown are the mean of 3 independent experiments ±SEM. See also Figures S11 and S12.

We first compared the effects of the three CK2 inhibitors on the endogenous CK2 activity in 786-O cells. As shown in Figure 3A (left panel), CK2 activity was strongly inhibited by micromolar concentrations of the three inhibitors. We next evaluated their effects on CK2-mediated downstream phosphorylation events by western blot analysis. Notably, CK2 is known to phosphorylate AKT at Ser129.^50^^,^^51^ As expected, AB668 as well as SGC-CK2-1 and CX-4945 decreased, in a dose-dependent manner, the phosphorylation of S129AKT (Figure 3A, right panel).

Previous work has demonstrated the anti-apoptotic role of the cyclin-dependent kinase inhibitor p21 in RCC (renal cell carcinoma) as a potential mechanism for their drug resistance.^52^ p21 binds to and inhibits the activity of proteins involved in apoptosis, including pro-caspase-3.^53^^,^^54^ Phosphorylation of p21 at Thr145 by AKT1 induces its cytoplasmic accumulation,^55^^,^^56^ and propels its anti-apoptotic functions.^53^^,^^54^ Interestingly, sorafenib, as one of the few available effective therapeutic options for metastatic RCC, was shown to attenuate the anti-apoptotic role of p21 in kidney cancer cells.^57^ As shown in Figure 3A, right panel, the AKT1-dependent phosphorylation of p21 was strongly downregulated by low concentrations of the three inhibitors, in accordance with their inhibitory effect on AKT phosphorylation in 786-O cells. Of note, we previously showed that CX-4945 inhibits the AKT1-dependent phosphorylation of p21 in 786-O cells.^19^

p38MAPK, a tumor suppressor well known for its role in transducing stress signals from the environment was activated to the same extent by the three inhibitors (Figure 3A). Interestingly, mouse modeling studies showed that mTOR activation in combination with inactivation of the p38MAPK initiates renal cell carcinoma.^58^ Altogether, this analysis highlights that several CK2 down-stream cellular events (AKT, p38MAPK, p21) are similarly modulated by the three inhibitors, at concentrations that correlate with their effect on in-cell CK2 activity (Figure 3A).

In contrast to SGC-CK2-1 and CX-4945, AB668 also induced a dose-dependent inhibition of the activated forms of STAT3, a protein that regulates proliferation and apoptosis in cancer cells.^59^ It was reported that inhibition of CK2 hinders STAT3 signaling and decreases aggressive phenotypes in multiple cancer types.^60^^,^^61^ Moreover, studies indicate that STAT3 activation plays a significant role in clear cell RCC and is associated with increased metastasis and worse survival outcomes.^62^^,^^63^

Finally, we have also evaluated the effects of AB668 in the triple-negative breast cancer cell line MDA-MB231 by western blot analysis. In comparison with 786-O cells, AB668 led to similar effects on CK2-mediated downstream phosphorylation events (Figures S11A and S11B). Of note, the viability of normal human breast epithelial cells (MCF10A) was not affected by high concentrations (20 μM) of AB668 (Figure S11A).

### Effects of AB668 on CK2 downstream events in healthy cells

Motivated by the striking difference in sensitivity to AB668 between cancer versus normal cells, we evaluated its target engagement by assaying the CK2 activity in extracts of human embryonic kidney HEK293 cells after treatment with increasing concentrations of AB668. IC_50_ values were 0.34 ± 0.07 μM and 0.60 ± 0.11 μM for 786-O cells and HEK293 cells, respectively (Figure 3B, left panel). These results show that AB668 inhibited CK2 activity with a similar potency in both cell types although it did not induce cytotoxicity in normal cells (Figures 2E and S10). Similarly, the viability of normal human breast epithelial cells (MCF10A) was not affected by high concentrations (20 μM) of AB668 (Figure S11A). Comparison of downstream phosphorylation events mediated by CK2 in 786-O and HEK293 cells, in response to AB668, indicates that p38 was activated in both cell types. However, in contrast to 786-O cells, AKT and p21 phosphorylation was weakly affected in HEK293 cells (Figure 3B, right panel). Of note, we noticed that the amount of total proteins (AKT and p21) was increased in response to AB668 treatment whereas at high concentration, p38 expression was downregulated.

### AB668 and CX-4945 induce differential transcriptome deregulation in cancer cells

**Figure 4:**
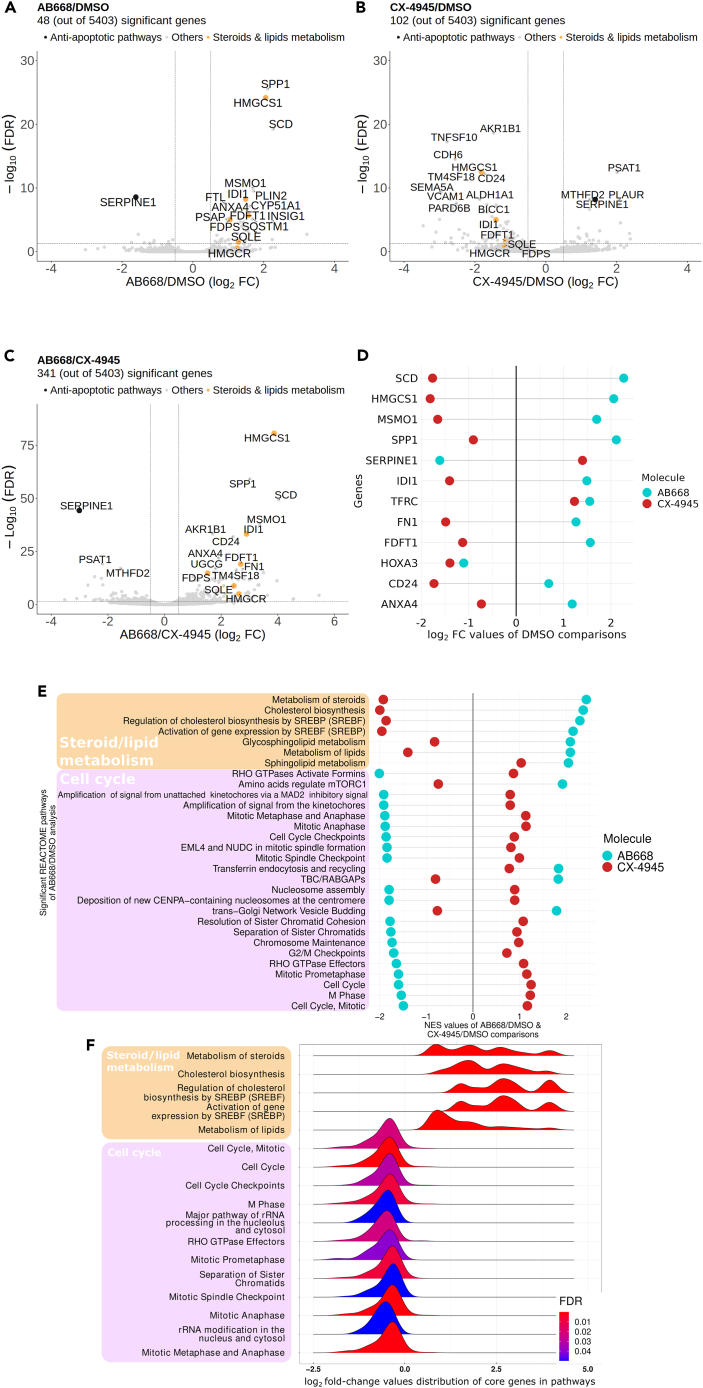
Deregulations of the transcriptome in response to AB668 or CX-4945 (A–C) Volcano plots based upon differential gene expression analysis of the transcriptomes of 786-O cells exposed to the different CK2 inhibitors: (A) AB668 vs*.* DMSO (B) CX-4945 vs. DMSO, and (C) AB668 vs*.* CX-4945. (D) Differences of Log2(Fold Changes) of gene expression values for AB668 and CX-4945 treated cells compared to DMSO treated cells. (E) Normalized enrichment scores (NES), calculated by the gene set enrichment analysis (GSEA) method, for significantly deregulated pathways obtained from AB668 vs*.* DMSO analysis in comparison to the NES scores generated from CX-4945 vs*.* DMSO analysis. (F) Histograms of Log2(Fold Changes) of gene expression values for AB668 vs*.* CX-4945 comparison for significantly deregulated pathways found by GSEA analysis, using REACTOME pathway database.

To assess the impact of AB668 and CX-4945 at the transcriptomic level, molecular profiling of 786-O cells treated with these CK2 inhibitors was performed by BRB-seq, followed by differential gene expression analysis (Figure 4A – left and center plot). Only 48 genes were significantly differentially expressed (Benjamini-Hochberg corrected p value <0.05 and |log_2_ (Fold change)| > 0.5) in response to AB668 when compared to cells treated with DMSO. In contrast, 102 genes were deregulated by CX-4945 compared to DMSO. This might be related to the lower selectivity of this CK2 inhibitor. Volcano plots and the DEG (Differencially expressed genes) numbers (341 DEGs for AB668 vs. CX-4945) on total genes show that a large proportion of genes (5062 genes out of 5403 genes) are not differentially expressed between the two molecular conditions used.

Since a large number of genes are not deregulated between the 2 molecules, this suggests that their influence on the transcriptome is rather similar. Nevertheless, 341 genes were significantly differentially expressed in cells exposed either to AB668 or CX-4945, revealing differences in transcriptome perturbations associated to these two CK2 inhibitors (Figure 4A—right plot). Interestingly, SERPINE1, also known as plasminogen activator inhibitor type-1 (PAI-1) a protein with a growth and migration stimulatory functions and an anti-apoptotic activity,^64^^,^^65^ was found significantly down-regulated by AB668, when compared to CX-4945 or DMSO. In ccRCC tumor tissues, the expression level of PAI-1 is higher than in normal tissues and has been proven to be a reliable biological and prognostic marker associated with poor prognosis.^66^ Metabolic enzymes such as phosphoserine aminotransferase (PSAT1), UDP-N-acetylglucosamine pyrophosphorylase 1 (UAP1), and methylenetetrahydrofolate deshydrogenase/cyclohydrolase (MTHFD2), that are highly expressed in a wide range of tumors and associated with poor prognosis in tumor progression,^67^^,^^68^^,^^69^^,^^70^^,^^71^ were significantly down regulated by AB668 when compared to CX-4945.

In addition, we specifically identified, among the genes differentially expressed compared to the DMSO control, those whose expression was deregulated in an opposite way between AB668 and CX-4945 (Figure 4B). For example, SERPINE1 is up-regulated by CX-4945 but down-regulated by AB668 compared to DMSO-exposed cells. Interestingly, genes involved in fatty acid, cholesterol and steroid metabolisms like, farnesyl-diphosphate farnesyltransferase 1 (FDFT1), 3-hydroxy-3-methylglutaryl-CoA synthase 1 (HMGCS1) and isopentenyl-diphosphate delta isomerase 1 (IDI1) were all up-regulated by AB668 while down-regulated by CX-4945. Other fatty acid metabolism related genes 3-hydroxy-3-methylglutaryl-CoA reductase (HMGCR) and squalene epoxidase (SQLE) were also up-regulated in response to AB668 compared to CX-4945 or DMSO (Figure 4A). This can be related to the recently pointed out role of CK2 in lipid homeostasis.^72^ CK2 was shown to regulate lipogenesis and adipogenesis at multiple levels. Moreover, the up-regulation of CK2 may help to sustain elevated rates of growth in malignant cells by controlling enzymes regulating key steps in the signaling pathways involved in lipogenesis.^72^ In particular, HIF (Hypoxia-Inducible factor) expression drives lipid deposition in ccRCC via the repression of fatty acid metabolism.^73^ Importantly, it was previously shown that low expression of FDPS, FDFT1, HMGCS1, HMGCR, and IDI1 genes and high expression of SQLE were associated with patients with high-risk ccRCC.^74^ Thus, by up-regulating 5 out of 6 genes in this prognostic signature, AB668 could represent a therapeutic option to counteract ccRCC tumor progression.

An identification of the biological pathways over-represented in genes deregulated in expression, in response to either AB668 or CX-4945 compared to DMSO, was then carried out using the gene set enrichment analysis (GSEA) method and the Reactome database. Among the 17 significantly altered pathways, 5 were related to the metabolism of steroids and lipids and 12 were connected to cell cycle and mitotic processes (Figure 4D). While pathways related to steroids and lipids metabolism were up-regulated in response to ABB668, cell cycle and mitotic processes-based pathways were down-regulated by AB668. This down-regulation of cell cycle is consistent with the strong inhibition of cell proliferation observed in cell lines treated with AB668. Furthermore, among the pathways identified as significantly deregulated in response to AB668 compared to DMSO, we found that all but two (transferin endocytosis and recycling and sphingolipid metabolism) have opposite deregulations in response to CX-4945 (Figure 4C), illustrating the strongly different effects of these CK2 inhibitors on these cellular processes.

In summary, our transcriptomic analysis clearly demonstrates that the inhibition of CK2 by AB668 or by the pure ATP-competitive CX-4945 inhibitor that reached clinical trials differentially affect biological pathways in 786-O cancer cells. More specifically, AB668 induces a deep alteration of antiapoptotic pathways, cell cycle and mitotic processes, as well as steroids and lipids metabolism, which all are involved in renal cancer tumorigenicity.

## Discussion

The key role of CK2-dependent pathways in cancer has motivated the development of CK2 inhibitors. While most of these inhibitors act by an orthosteric mechanism, meaning that they bind to the highly conserved ATP binding-pocket of the kinase,^75^ small molecules that act outside the ATP site have been also described.^31^ In particular, two bivalent CK2 inhibitors targeting the ATP site and the αD pocket have been previously published with the aim to design highly selective CK2 inhibitors.^33^^,^^35^

Here, we have disclosed a bivalent CK2 inhibitor that binds at both the ATP site and the allosteric αD pocket, a feature that accounts for its high selectivity profile in the human kinome, as previously reported for two other CK2 bivalent inhibitors.^33^^,^^35^

The presence of this ligandable allosteric pocket on CK2 was previously revealed during a crystallographic fragment screening campaign,^33^ in agreement with the high mobility of the αD helix in CK2. The flexibility of this αD helix was experimentally observed in various crystallographic structures of the kinase and was also reported in metadynamic studies of CK2 structure.^76^ Because of the unique plasticity of the CK2 helix αD, inhibitors targeting the αD pocket exhibit an extraordinary selectivity among other protein kinases. Interestingly, a computational study looking for kinases allosteric sites did not predict the αD pocket as an allosteric pocket in other kinases.^77^

In order to use AB668 as a chemical tool to explore further in-cell CK2 function, we first examined the activity of AB668 in cancer and healthy cells. Treatment with AB668 strongly impacted cancer cell viability resulting in apoptotic cell death, while sparing healthy cells. This observation was in agreement with the literature, as a long-held view has suggested that malignant cells are dependent upon sustained CK2 signaling for survival thereby exhibiting an exquisite sensitivity to CK2 inhibition.^21^^,^^22^^,^^23^ The controversial finding that CX-4945 does not affect cell viability of renal cancer cells is in agreement with our previous observations.^78^ Conversely, non-cancer cells are more resistant to induction of cell death upon downregulation of CK2 activity, which is the expected basis for safely using a pharmacological chemical inhibitor.

To date, the cytotoxicity of inhibitors targeting the helix αD has been reported, but no study exploring their impact on CK2-dependent cellular pathways has been published. Here, we have compared the effects of the bivalent CK2 inhibitor AB668 to CX-4945 and SGC-CK2-1 using caspase activation assay, live-cell imaging, and transcriptomic analysis. Our data highlight that AB668 has a distinct mechanism of action regarding its anti-cancer activity (Figures 2, 3, and 4). These observations suggest that targeting the allosteric CK2 αD pocket has a distinct cellular impact than targeting only the CK2 ATP binding site. One hypothesis is that, by inserting into the αD pocket, bivalent inhibitors such as AB668 may differentially affect the recognition of different CK2 substrates or might impact non-catalytic activities through the perturbation of interactions between CK2 and key cellular proteins. This is consistent with a recent phosphoproteomic study where different CK2 substrates have been identified by enrichment analysis of SGC-CK2-1- and CX-4945- dependent phosphoproteomes.^79^ Further studies will be required to characterize the mechanism of action of AB668, since we cannot exclude that AB668 might have CK2-independent effects due to interactions with off-targets outside the kinome.

Transcriptomic analysis highlights striking differences in biological pathways induced by cell treatment with either AB668 or the ATP-competitive inhibitor CX-4945. AB668 acts by an unconventional mechanism and induces strong apoptotic cell death. Thus, this strong pharmacological effect observed in various functional assays indicates that AB668 is an important investigational probe for exploiting apoptotic vulnerabilities in cancer as well as a promising lead for the next generation of drug-like CK2 inhibitors with improved potency and optimal drug properties. Future experiments will need to define further the mechanisms by which AB668, by occupying the allosteric αD pocket, induces apoptotic cell death in cancer cells, while sparing healthy cells. Taken together, our results strongly suggest that CK2 inhibition using small molecules that target binding sites outside the ATP pocket could be a valuable strategy in treating various aggressive cancers and disease-relevant contexts.

### Limitations of the study

Our study describes a bivalent inhibitor for CK2, a protein kinase that is highly dysregulated, modulating all cancer hallmarks. As compared to SGC-CK2-1 and CX-4945, AB668 affects cancer cell viability. STAT3 activation plays a key role in RCC and is associated with increased metastasis and worse survival outcomes.^62^^,^^63^ Interestingly, analysis of CK2 downstream events showed that the STAT3 signaling pathway was only impaired by AB668. However, more studies are warranted to explore the connection and regulatory mechanisms between AB668-mediated CK2 inhibition and cell death. Further studies will be required to characterize the mechanism of action of AB668, since we cannot exclude that AB668 might have CK2-independent effects due to interactions with off-targets outside the kinome. We also highlighted the striking difference in sensitivity to AB668 between cancer versus normal cells. Comparison of downstream phosphorylation events mediated by CK2 in response to AB668, showed that AKT and p21 phosphorylation was weakly affected in normal renal cells in contrast to 786-O cells. We need to work on it in the future rising ADMET data. Moreover, we showed the efficacy of AB668 on an *ex vivo* culture model of renal carcinoma. However, to better understand the anti-cancer effect of AB668 experiments in mice should be performed.

Overall, our study illustrates that AB668 provides insightful guidance for the next generation of drug-like CK2 inhibitors with improved potency and optimal drug properties in cancer-relevant contexts.

## STAR★Methods

### Key resources table

**Table:** 

REAGENT or RESOURCE	SOURCE	IDENTIFIER
**Antibodies**		

GAPDH, Mouse monoclonal antibody	ThermoFisher Scientific	Thermo Fisher Scientific Cat# AM4300, RRID:AB_2536381
P-AKT-phospho-Ser129, Rabbit polyclonal antibody	Interchim (Abgent)	Abgent Cat# AP3020a, RRID:AB_2289403
AKT, Rabbit polyclonal antibody	Cell Signaling Technology	Cell Signaling Technology Cat# 9272, RRID:AB_329827
PARP, Rabbit polyclonal antibody	Cell Signaling Technology	Cell Signaling Technology Cat# 9542, RRID:AB_2160739
mTOR, Rabbit polyclonal antibody	Cell Signaling Technology	Cell Signaling Technology Cat# 2972, RRID:AB_330978
Phospho-mTOR (Ser2448), Rabbit polyclonal Antibody	Cell Signaling Technology	Cell Signaling Technology Cat# 2971, RRID:AB_330970
p53, Rabbit polyclonal antibody	Cell Signaling Technology	Cell Signaling Technology Cat# 9282, RRID:AB_331476
Phospho-p53 (Ser15) (16G8) Mouse mAb antibody	Cell Signaling Technology	Cell Signaling Technology Cat# 9286, RRID:AB_331741
p38 MAPK, Rabbit polyclonal antibody	Cell Signaling Technology	Cell Signaling Technology Cat# 9212, RRID:AB_330713
p38MAPK-phospho-Thr180/Tyr182, Rabbit polyclonal antibody	Cell Signaling Technology	Cell Signaling Technology Cat# 9211, RRID:AB_331641
Stat3 (79D7), Rabbit monoclonal antibody	Cell Signaling Technology	Cell Signaling Technology Cat# 4904, RRID:AB_331269
STAT3-phospho-Ser727, Rabbit polyclonal antibody	Cell Signaling Technology	Cell Signaling Technology Cat# 9134, RRID:AB_331589
Survivin, Rabbit polyclonal antibody	Novus biologicals	Novus Cat# NB500-201, RRID:AB_10001517
p21 (C19), Rabbit polyclonal antibody	Santa Cruz Biotechnology	Santa Cruz Biotechnology Cat# sc-397, RRID:AB_632126
p21-phospho-Thr145, Rabbit polyclonal antibody	Abcam	Abcam Cat# ab47300, RRID:AB_881828
Serpine1, Goat polyclonal antibody	Antibodies	Antibodies.com Cat# A84079, RRID:AB_2187048
peroxidase-conjugated affinity pure Goat anti-rabbit IgG	Jackson ImmunoResearch Labs	Jackson ImmunoResearch Labs Cat# 111-035-003, RRID:AB_2313567
Peroxidase-AffiniPure Goat Anti-Mouse IgG	Jackson ImmunoResearch Labs	Jackson ImmunoResearch Labs Cat# 115-035-003, RRID:AB_10015289
Anti-Casein Kinase II Substrate, Phospho-Specific (9F4), mouse monoclonal antibody	Millipore	Millipore Cat# 231576-100UG, RRID:AB_211721

**Chemicals, peptides, and recombinant proteins**		

CX-4945	SelleckChem	1009820-21-6
SGC-CK2-1	Sigma-Merck	2470424-39-4
staurosporine	Sigma-Merck	62996-74-1
human recombinant CK2α subunit	Hériché et al.^80^	CK2α
MBP (maltose-binding protein)-CK2β	Chantalat et al.^81^	CK2β
GST(Glutathion-S-Transferase)-SIX1	https://novoprolabs.com/p/human-six1-recombinant-protein-gst-tag-524304.html	Cat.#: 524304
SYPRO Orange	ThermoFisher Scientific/Invitrogen	Cat#: S6650
DMEM medium Gibco™	ThermoFisher Scientific	Cat#:32430100
ProXup medium	Evercyte	Cat#: MHT-003-2 / MHT-003-2-B + MHT-003-S
Phosphatase inhibitor cocktail	Sigma-Merck	Cat#: P8340, P2850, P5726
2-chloroethylamine hydrochloride	Sigma-Aldrich	CAS 870-24-6
4-isobutylbenzenesulfonyl chloride	Fischer Scientific	CAS 339370-45-5
acetonitrile	Fischer Scientific	CAS 75-05-8
acetyl chloride	Sigma-Aldrich	CAS 75-36-5
bis(triphenylphosphine)palladium(II) dichloride	Sigma-Aldrich	CAS 13965-03-2
copper(I) iodide	Alfa Aesar	CAS 7681-65-4
Cyclohexane	Fischer Scientific	*CAS. 110-82-7*
Dichloromethane	Fischer Scientific	*CAS. 75-09-2*
diethyl ether	Fischer Scientific	*CAS 60-29-7*
diisopropyl azodicarboxylate	Fluorochem	*CAS 2446-83-5*
ethyl acetate	Fischer Scientific	*CAS 141-78-6*
Isobutanol	Merck	*CAS. 78-83-1*
Methanesulfonyl chloride	Sigma-Aldrich	*CAS 124-63-0*
methanol	Fischer Scientific	*CAS 67-56-1*
MgSO_4_	Sigma-Aldrich	*CAS 7487-88-9*
N,*N*-diisopropylethylamine	Sigma-Aldrich	*CAS 7087-68-5*
*N*,*N*-dimethylformamide	Sigma-Aldrich	*CAS. 68-12-2*
potassium carbonate	Fischer Scientific	*CAS 584-08-7*
potassium hydroxide	Fischer Scientific	CAS 1310-58-3
sodium ascorbate	Alfa Aesar	*CAS 134-03-2*
sodium azide	Sigma-Aldrich	*CAS 26628-22-8*
sodium bisulfite	Sigma-Aldrich	*CAS 7631-90-5*
tetrabutylammonium fluoride	Sigma-Aldrich	*CAS* 429-41-4
tetrahydrofuran	Fischer Scientific	*CAS 109-99-9*
*tert*-butanol	Acros	CAS 75-65-0
triethylamine	Sigma-Aldrich	*CAS 121-44-8*
trimethylsilylacetylene	Sigma-Aldrich	*CAS 1066-54-2*
Triphenylphosphine	Fischer Scientific	*CAS 603-35-0*

**Critical commercial assays**		

Incucyte zoom	Sartorius	Essen Incucyte Incucyte (RRID:SCR_019874)
CellEvent™ Caspase-3/7 Green Detection Reagent	ThermoFisher Scientific	C10740
MirVana PARIS kit	ThermoFisher Scientific	AM1556
Caspase-3 Fluorometric Assay Kit II	Biovision/abcam	ab252897.

**Deposited data**		

ArrayExpress Annotare 2.0	https://www.ebi.ac.uk/fg/annotare/	E-MTAB-12776
Protein structure deposition at PDB	https://www.rcsb.org	8C5Q

**Experimental models: Cell lines**		

786-O	ATCC	RRID:CVCL_1051
HEK293T	ATCC	RRID:CVCL_0063
MDA-MB231	ATCC	RRID:CVCL_0062
RPTEC	Evercyte	RRID:CVCL_K278
A375	ATCC	RRID:CVCL_0132
A549	ATCC	RRID:CVCL_0023

**Experimental models: Organisms/strains**		

Six week-old BALB/c Female nude mice	Charles River Laboratories	Cat#: 194NU/NUB/C

**Software and algorithms**		

GraphPad Prism 9.2.0	GraphPad Software, Inc.	http://www.graphpad.com
IncucyteZoom	Sartorius	Zoom
Bioconductor/R package clusterProfiler 4.6.0		https://www.essenbioscience.com/en/resources/incucyte-zoom-resources-support/
ImageJ, v1.52	National Institutes of Health software	https://imagej.nih.gov/ij/
Coot		https://www2.mrc-lmb.cam.ac.uk/personal/pemsley/coot/
Phenix		https://phenix-online.org
Grade server		http://grade.globalphasing.org/cgi-bin/grade/server.cgi
TSA-CRAFT		https://sourceforge.net/projects/tsa-craft

### Resource availability

#### Lead contact

Further information and requests for resources and reagents should be directed to and will be fulfilled by the lead contact, Isabelle Krimm (isabelle.krimm@univ-lyon1.fr).

#### Materials availability

This study report new unique material (the CK2 inhibitor AB668) that is patented and there are consequently restrictions to availability.

#### Data and code availability

• Transcriptomic data have been deposited at arrayexpress and are publicly available as of the date of publication. https://www.ebi.ac.uk/biostudies/arrayexpress/studies/E-MTAB-12776. Crystallographic data have been deposited at the PDB under the PDB ID 8C5Q.
• This paper does not report original code.
• Any additional information required to reanalyse the data reported in the paper is available from the lead contact upon request.

### Experimental model and study participant details

#### Cell lines and cell culture

All cell lines were purchased from American Type Culture Collection (ATCC) and grown on standard tissue culture plastic in a 5% CO_2_ humidified incubator at 37°C. 786-O were maintained in RPMI 1640 medium (Gibco), containing 10% of FBS, penicillin (24 U/mL), and streptomycin (25 μg/mL). A549, A375 and MDA-MB231 were cultured in DMEM + GlutaMAX medium (Gibco) supplemented with 10% of FBS. HEK293T were grown in EMEM supplemented with 10% of FBS and RPTEC were maintained in ProXup (Evercyte). MCF10A were cultured as described.^82^

#### *In vivo* orthotopic tumor xenograft models

All animal studies were approved by the institutional guidelines and those formulated by the European Community for the Use of Experimental Animals. Six-week-old BALB/c Female nude mice (Charles River Laboratories) with a mean body weight of 18-20 g were used to establish orthotopic xenograft tumor models. The mice were housed and fed under specific pathogen-free conditions. To produce tumors, renal cancer cells 786-O-luc were harvested from subconfluent cultures by a brief exposure to 0.25 % trypsin-EDTA. Trypsinization was stopped with medium containing 10 % FBS, and the cells were washed once in serum-free medium and resuspended in 500 μl PBS. Renal orthotopic implantation was carried out by injection of 3 × 10^6^ 786-O luc cells into the right kidney of athymic nude mice. Mice were weighed once a week to monitor their health and tumor growth was measured by imaging luminescence of 786-O-luc cells (IVIS).

#### Chemistry

All commercially available chemicals and solvents were purchased and were used received unless otherwise stated. All reactions were carried out under argon atmosphere in flame-dried glassware as indicated and the reaction progress was monitored qualitatively using thin layer chromatography (TLC) aluminium plates precoated with Merck silica gel 60 F254. The spots were detected with UV light (254 nm or 356 nm) or by staining with ninhydrin (2% solution). The purification of products was performed on silica gel 60 (particle size 0.040 - 0.063 mm) from Merck 9385 Kieselgel using flash technique and under a positive pressure. The crude mixtures were adsorbed on silica gel 60 (particle size 0.040 - 0.063 mm) from Merck 9385 Kieselgel before chromatographic purification. Nuclear magnetic resonance spectra (NMR) were recorded on Bruker Avance 400 (400 MHz) and Bruker Avance 500 (500 mHz) spectrometers. Chemical shifts (à) are referenced to the solvent residual peak and are quoted in parts per million (ppm) to the nearest 0.01 ppm for 1H and to the nearest 0.1 ppm for 13C. d6-Acetone, CDCl3 and d6-DMSO were used as deuterated solvents and the resonances were locked as internal standards (d_6_-Acetone ^1^H δ = 2.05, ^13^C δ = 29.8 ; CDCl_3_^1^H δ = 7.26, ^13^C δ = 77.1 and d_6_-DMSO ^1^H à = 2.50, ^13^C δ = 39.5). The multiplicity of the signals is indicated by lower-case letters (s singlet, d doublet, t triplet, q quadruplet, m multiplet or overlap of non- equivalent resonances, br broad, br s singlet, or combination of letters) and coupling constants (J) are reported in Hertz to the nearest 0.1 Hz. Carbon multiplicity was determined by DEPT 135 experiments. Yields refer to isolated compounds, estimated to be > 98% pure as determined by ^1^H NMR or by high-performance liquid chromatography (HPLC). Liquid chromatography analyses were carried out Agilent 1290 Infinity system (Agilent Technologies) and chromatographic separations were performed on a reversed phase column Poroshell 120 SB-C18 Agilent (50 mm x 2.1 mm/ 2.7 μm). High-resolution mass spectra (HRMS) were recorded on a Bruker Micromass Q-TOF spectrometer using electrospray ionization (ESI). Melting points data (Mp) were collected on a Büchi B-545 and are uncorrected.

**Figure:**
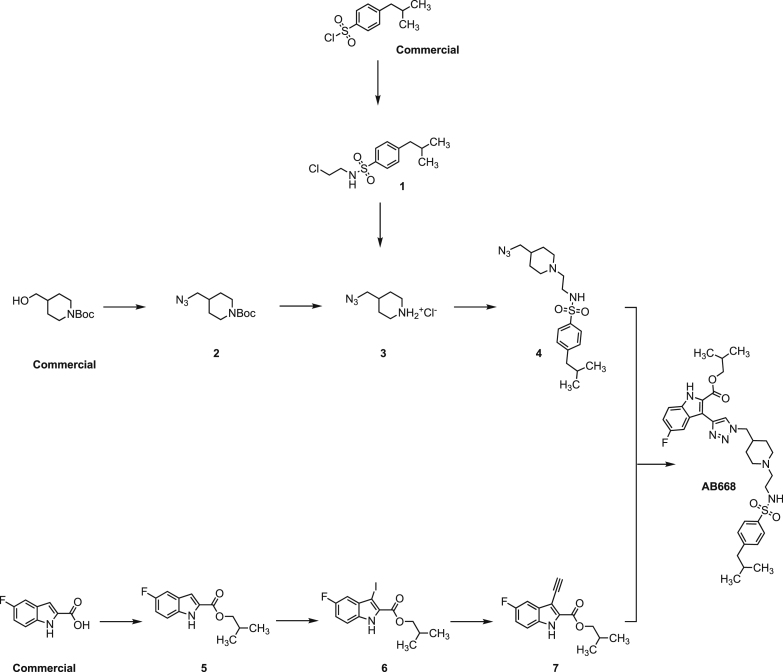

**Chemical synthesis scheme for AB668** (isobutyl 5-fluoro-3-(1-((1-(2-((4-isobutylphenyl)sulfonamido)ethyl)piperidin-4-yl)methyl)-1*H*-1,2,3-triazol-4-yl)-1*H*-indole-2-carboxylate). **Details of conditions and yields for each step are presented below.**

### Method details

#### Synthesis of compound 1: *N*-(2-chloroethyl)-4-isobutylbenzenesulfonamide

In an oven-dried round bottom flask was added at 10°C (ice bath) 2-chloroethylamine hydrochloride (500 mg, 4.31 mmol, 1.00 equiv.), potassium carbonate (1.20 g, 8.62 mmol, 1.00 equiv.) and 4-isobutylbenzenesulfonyl chloride (1.00 g, 4.31 mmol, 1.00 equiv.) in dichloromethane - water (15 mL, 2:1, C ∼ 0.3 M). The reaction was stirred for 3 h at 10 ± 3°C then allowed to warm up at room temperature and stirred for 12 h (monitored by TLC). After completion, the pH value of the reaction was adjusted around 7-8 with a slow addition of potassium carbonate (1.20 g, 8.62 mmol, 1.00 equiv.). The mixture layers were partitioned and extracted with dichloromethane three times (3 x 100 mL). The combined organic extracts were washed with brine (100 mL), dried (MgSO_4_) and filtered. The solvents were removed under reduced pressure and the residue was purified chromatographically on silica gel (eluting cyclohexane - ethyl acetate 85:15) to provide *N*-(2-chloroethyl)-4-isobutylbenzenesulfonamide as a white solid (835 mg, 70% yield).

#### *N*-(2-chloroethyl)-4-isobutylbenzenesulfonamide

^**1**^**H NMR** (400 MHz, d_6_-DMSO) δ = 7.91 (t, *J* = 5.9 Hz, 1H), 7.72 (d, *J* = 8.3 Hz, 2H), 7.46 (d, *J* = 8.3 Hz, 2H), 3.56 (t, *J* = 6.2 Hz, 2H), 3.06 (q, *J* = 6.1 Hz, 2H), 1.48-1.35 (m, 1H), 1.10 (qd, *J* = 12.1, 3.8 Hz, 2H), 0.91 (d, *J* = 6.7 Hz, 6H). ^**13**^**C NMR** (100 MHz, d_6_-DMSO) δ = 144.5 (C_quat_), 136.3 (C_quat_) 129.5 (CH), 126.4 (CH), 44.2 (CH_2_), 43.6 (CH_2_), 29.5 (CH), 29.1 (CH_2_), 22.0 (CH_3_). **LC-MS** Found: [M+H]^+^, 271.1.

#### Synthesis of compound 2: tert-butyl 4-(azidomethyl)piperidine-1-carboxylate

In an oven-dried round bottom flask were combined under argon atmosphere at 0°C (ice bath) *tert*-butyl 4-(hydroxymethyl)piperidine-1-**c**arboxylate (18.0 g, 83.6 mmol, 1.00 equiv.) and triethylamine (15.2 mL, 108.7 mmol, 1.30 equiv.) in dry tetrahydrofuran (250 mL, C ∼ 0.3 M). Methanesulfonyl chloride (6.47 mL, 83.6 mmol, 1.00 equiv.) was added dropwise through a syringe over 10 minutes then the reaction was allowed to warm to room temperature and stirred for 3 h. After the complete consumption of the starting material (monitored by TLC), the mixture was poured into ethyl acetate (400 mL) and water (250 mL). The organic layer was extracted, washed with 10% aqueous hydrochloric acid solution (150 mL), brine (150 mL), dried (MgSO_4_) and filtered. The solvents were removed under reduced pressure to obtain the product (23.6 g, 96% yield) as a white solid used without further purification for the next step. In an oven-dried round bottom flask equipped with a reflux condenser was dissolved the *tert*-butyl 4-(((methylsulfonyl)oxy)-methyl)piperidine-1-carboxylate (20.0 g, 68.2 mmol, 1.00 equiv.) in *N*,*N*-dimethylformamide (450 mL, C ∼ 0.15 M) and sodium azide (13.3 g, 0.2 M, 3.00 equiv.) was added in portion wise. The reaction mixture was heated to 60°C and stirred for 8 h until the complete consumption of the starting material (monitored by TLC). The reaction was allowed to cool to room temperature, poured into water (800 mL) and extracted with ethyl acetate (3 x 200 mL). The combined organics extracts were washed with water (3 x 200 mL), brine (100 mL), dried (MgSO_4_) and filtered. The solvents were removed under reduced pressure and the residue was purified chromatographically on silica gel (eluting cyclohexane - ethyl acetate 90:10) to provide *tert*-butyl 4-(azidomethyl)piperidine-1-carboxylate as a colorless oil (15.6 g, 95% yield, 91% total yield over two steps).

#### tert-butyl 4-(azidomethyl)piperidine-1-carboxylate

^**1**^**H NMR** (400 MHz, CDCl_3_) δ = 4.13 (bs, 2H), 3.19 (d, *J* = 6.3 Hz, 2H), 2.69 (bs, 2H), 1.73-1.66 (m, 3H), 1.45 (s, 9H), 1.24-1.10 (m, 2H). ^**13**^**C NMR** (100 MHz, CDCl^3^) δ = 154.6 (C_quat_), 79.3 (C_quat_), 56.9 (CH_2_), 43.7 (CH_2_), 42.9 (CH), 36.4 (CH_2_), 29.5 (CH_2_), 28.3 (CH_3_). **LC-MS** Found : [M+H]^+^, 241.1. Spectroscopic and physical data matched the ones reported in the literature (Loudet et al., 2008).

#### Synthesis of compound 3: 4-(azidomethyl)piperidine hydrochloride

In an oven-dried round bottom flask was dissolved *tert*-butyl 4-(azidomethyl)piperidine-1-carboxylate (15.2 g, 63.3 mmol, 1.00 equiv.) in methanol (210 mL, C ∼ 0.2 M). The solution was cooled to 0°C and acetyl chloride (54.2 mL, 760 mmol, 12.0 equiv.) was added dropwise through a syringe over 30 minutes. The reaction was allowed to warm to room temperature and stirred for 18 h until the complete consumption of the starting material (monitored by TLC). Excess of acetyl chloride and methanol were removed under reduced pressure and the residue was poured into diethyl ether and stirred at room temperature for 1 h. The resulting precipitate was filtered off, washed with cold diethyl ether (3 x 100 mL) and dried under reduced pressure to provide 4-(azidomethyl)piperidine hydrochloride as a white cristalline solid (11.0 g, 98% yield).

#### 4-(azidomethyl)piperidine hydrochloride

^**1**^**H NMR** (400 MHz, d_6_-DMSO) δ = 3.36 (s, 1H), 3.30 (d, *J* = 6.2 Hz, 2H), 3.21 (d, *J* = 12.6 Hz, 2H), 2.81 (t, *J* = 12.2 Hz, 2H), 1.77 (d, *J* = 12.7 Hz, 3H), 1.42 (qd, *J* = 13.5, 3.9 Hz, 2H). ^**13**^**C NMR** (100 MHz, d_6_-DMSO) δ = 55.4 (CH_2_), 42.4 (CH_2_), 33.4 (CH), 25.7 (CH_2_). **LC-MS** Found: [M+H]^+^, 141.0.

#### Synthesis of compound 4: *N*-(2-(4-(azidomethyl)piperidin-1-yl)ethyl)-4-isobutylbenzenesulfonamide

In an oven-dried round bottom flask equipped with a reflux condenser and under argon atmosphere was dissolved the ammonium salt **3** (300 mg, 1.70 mmol, 1.00 equiv.) in dry acetonitrile (15 mL, C ∼ 0.15 M) then treated with *N*,*N*-diisopropylethylamine (887 μL, 5.10 mmol, 3.00 equiv.), added slowly through a syringe. The mixture was stirred for 10 minutes at room temperature before the addition in portion wise of compound **1** (515 mg, 1.87 mmol, 1.10 equiv.) and a catalytic amount of potassium iodide. The reaction was heated to 82°C (preheated oil bath) for 18 h. After the complete consumption of the starting material (monitored by TLC), the mixture was allowed to cool to room temperature and then poured into water and extracted with dichloromethane three times (3 x 50 mL). The combined organic extracts were washed with brine (100 mL), dried (MgSO_4_) and filtered. The solvents were removed under reduced pressure and the residue was purified by column chromatography on silica gel (eluting gradient dichloromethane - methanol 98:2 to 96:4) to provide *N*-(2-(4-(azidomethyl)piperidin-1-yl)ethyl)-4-isobutylbenzenesulfonamide as a colorless oil (461 mg, 71% yield).

#### *N*-(2-(4-(azidomethyl)piperidin-1-yl)ethyl)-4-isobutylbenzenesulfonamide

^**1**^**H NMR** (400 MHz, d_6_-DMSO) δ = 7.75-7.67 (m, 2H), 7.42-7.33 (m, 3H), 3.20 (d, *J* = 6.6 Hz, 2H), 2.83 (dt, *J* = 8.6, 4.3 Hz, 2H), 2.66 (dd, *J* = 11.1, 4.0 Hz, 2H), 2.52 (d, *J* = 7.1 Hz, 2H), 2.24 (dd, *J* = 7.6, 6.2 Hz, 2H), 1.93-1.74 (m, 3H), 1.58-1.49 (m, 2H), 1.48-1.35 (m, 1H), 1.11 (qd, *J* = 12.1, 3.9 Hz, 2H), 0.86 (d, *J* = 6.6 Hz, 6H). ^**13**^**C NMR** (100 MHz, d_6_-DMSO) δ = 145.9 (C_quat_), 138.1 (C_quat_), 129.5 (CH), 126.4 (CH), 56.9 (CH_2_), 56.2 (CH_2_), 52.7 (CH_2_), 44.2 (CH_2_), 40.2 (CH_2_), 35.6 (CH), 29.5 (CH), 29.1 (CH_2_), 22.0 (CH_3_). **LC-MS** Found : [M+H]^+^, 380.2.

#### Synthesis of compound 5: Isobutyl 5-fluoro-1*H*-indole-2-carboxylate

In an oven-dried round bottom flask was placed under argon atmosphere 5-fluoro-1*H*-indole-2-carboxylic acid-2-carboxylic acid (2.50 g, 13.9 mmol, 1.20 equiv.), triphenylphosphine (3.66 g, 13.9 mmol, 1.20 equiv.) and isobutanol (1.08 mL, 11.6 mmol, 1.00 equiv.) in dry tetrahydrofuran (23 mL, C ∼ 0.5 M). The reaction mixture was cooled to 0°C and diisopropyl azodicarboxylate (2.75 mL, 13.9 mmol, 1.20 equiv.) was added dropwise through a syringe over 10 minutes. The reaction was allowed to warm up to room temperature and stirred for 48 h until the complete consumption of the starting material (monitored by TLC). The solvents were removed under reduced pressure and the crude was passed through a short silica gel column eluting with diethyl ether - dichloromethane (1:1) then purified on silica gel (eluting cyclohexane - ethyl acetate 90:10) to provide isobutyl 5-fluoro-1*H*-indole-2-carboxylate as a pale-yellow solid (2.78 g, 85% yield).

#### Isobutyl 5-fluoro-1*H*-indole-2-carboxylate

^**1**^**H NMR** (400 MHz, CDCl_3_) δ = 9.12 (s, 1H), 7.37 (dd, *J* = 9.0, 4.4 Hz, 1H), 7.32 (dd, *J* = 9.2, 2.4 Hz, 1H), 7.19 (dd, *J* = 2.1, 0.9 Hz, 1H), 7.09 (td, *J* = 9.1, 2.5 Hz, 1H), 4.16 (d, *J* = 6.7 Hz, 2H), 2.10 (dq, *J* = 13.4, 6.7 Hz, 1H), 1.04 (d, *J* = 6.7 Hz, 6H). ^**13**^**C NMR** (100 MHz, CDCl_3_) δ = 162.1 (C_quat_), 158.3 (d, *J* = 236.6 Hz, C_quat_), 133.6 (C_quat_), 129.14 (C_quat_), 127.8 (d, *J* = 10.4 Hz, CH), 114.6 (d, *J* = 27.1 Hz, CH), 113.0 (d, *J* = 9.5 Hz, CH), 108.5 (d, *J* = 5.0 Hz, CH), 106.8 (d, *J* = 23.3 Hz, C_quat_), 71.3 (CH_2_), 21.1 (CH), 19.3 (CH_3_). ^**19**^**F NMR** (376 MHz, d_6_-DMSO) δ = -121.5. **LC-MS** Found: [M+H]^+^, 236.1.

#### Synthesis of compound 6: Isobutyl 5-fluoro-3-iodo-1*H*-indole-2-carboxylate

In an oven-dried round bottom flask was dissolved the isobutyl 5-fluoro-1*H*-indole-2-carboxylate (2.70 g, 11.48 mmol, 1.00 equiv.) in *N*,*N*-dimethylformamide (19 mL, C ∼ 0.6 M) and treated with potassium hydroxide (2.25 g, 40.17 mmol, 3.50 equiv.) during 10 minutes at room temperature. Then a solution of diiode (2.94 g, 11.60 mmol, 1.01 equiv.) in *N*,*N*-dimethylformamide (16.5 mL, C ∼ 0.7 M) was added dropwise through a syringe and the mixture was stirred at room temperature for 4 h until the complete consumption of the starting material (monitored by TLC). The reaction mixture was then poured into ice water (C ∼ 0.1 mM) containing 0.5% sodium bisulfite and 2.5% ammonia. The solution was placed in a refrigerator to ensure the complete precipitation then the resulting precipitate was filtered off, washed with ice water. The precipitate was dried at 50°C under reduced pressure during 72 h. Isobutyl 5-fluoro-3-iodo-1*H*-indole-2-carboxylate (3.82 g, 92% yield) was used without further purification for the next step.

#### Isobutyl 5-fluoro-3-iodo-1*H*-indole-2-carboxylate

^**1**^**H NMR** (400 MHz, d_6_-DMSO) δ = 12.34 (s, 1H), 7.51 (dd, *J* = 9.0, 4.5 Hz, 1H), 7.21 (td, *J* = 9.2, 2.5 Hz, 1H), 7.14 (dd, *J* = 9.4, 2.4 Hz, 1H), 4.13 (d, *J* = 6.4 Hz, 2H), 2.08 (dp, *J* = 13.2, 6.6 Hz, 1H), 1.04 (d, *J* = 6.7 Hz, 6H). ^13^C NMR (100 MHz, d_6_-DMSO) δ = 160.4 (C_quat_), 158.0 (d, *J* = 235.8 Hz, C_quat_), 133.6 (C_quat_), 131.0 (d, *J* = 10.4 Hz, C_quat_), 128.9 (C_quat_), 114.9 (d, *J* = 34.9 Hz, CH), 114.8 (d, *J* = 1.80 Hz, CH), 106.6 (d, *J* = 23.8 Hz, CH), 70.9 (CH_2_), 64.8 (d, *J* = 5.5 Hz, C_quat_), 27.4 (CH), 19.1 (CH_3_). ^19^F NMR (376 MHz, d_6_-DMSO) δ = -121.5. LC-MS Found: [M+H]^+^, 361.9.

#### Synthesis of compound 7: Isobutyl 3-ethynyl-5-fluoro-1*H*-indole-2-carboxylate

In an oven-dried round bottom flask equipped with a reflux condenser and under argon atmosphere, isobutyl 5-fluoro-3-iodo-1*H*-indole-2-carboxylate (3.70 g, 10.25 mmol, 1.00 equiv.), bis(triphenylphosphine)palladium(II) dichloride (360 mg, 0.51 mmol, 5 mol%) and copper(I) iodide (195 mg, 1.02 mmol, 10 mol%) were combined in dry tetrahydrofuran (50 mL, C ∼ 0.2 M). The reaction mixture was degassed with argon over 5 minutes before a slowly addition of trimethylsilylacetylene (2.13 mL, 15.4 mmol, 1.50 equiv.) and dry triethylamine (7.14 mL, 51.2 mmol, 5.00 equiv.). The solution was then heated to 60°C (preheated oil bath) for 18 h (monitored by TLC). The mixture was allowed to cool to room temperature and 1M solution of tetrabutylammonium fluoride in tetrahydrofuran (15.4 mL, 15.4 mmol, 1.50 equiv.) was added dropwise through a syringe over 10 minutes and stirred for 0.5 h until the complete deprotection (monitored by TLC). The resulting solution was quenched by addition of a saturated solution aqueous of ammonium chloride (100 mL) and extracted with ethyl acetate three times (3 x 100 mL). The combined organic extracts were washed with brine (100 mL), dried (MgSO_4_) and filtered. The solvents were removed under reduced pressure and the residue was purified chromatographically on silica gel (eluting cyclohexane - ethyl acetate 90/10) to provide **isobutyl 3-ethynyl-5-fluoro-1*H*-indole-2-carboxylate** as a pale yellow solid (1.71 g, 65% yield).

#### Isobutyl 3-ethynyl-5-fluoro-1*H*-indole-2-carboxylate

^**1**^**H NMR** (400 MHz, d_6_-DMSO) δ = 12.78 (s, 1H), 8.02-7.94 (m, 1H), 7.81 (dd, *J* = 9.1, 2.6 Hz, 1H), 7.69 (td, *J* = 9.2, 2.6 Hz, 1H), 4.93 (s, 1H), 4.60 (d, *J* = 6.3 Hz, 2H), 2.50 (h, *J* = 6.6 Hz, 1H), 1.48 (d, *J* = 6.7 Hz, 6H). ^**13**^**C NMR** (100 MHz, d_6_-DMSO) δ = 160.3 (C_quat_), 158.0 (d, *J* = 237.3 Hz, C_quat_), 132.6 (C_quat_), 130.5 (C_quat_), 129.0 (d, *J* = 10.8 Hz, C_quat_), 114.8 (d, *J* = 10.9 Hz, CH), 114.6 (d, *J* = 6.2 Hz, CH), 104.6 (d, *J* = 23.9 Hz, CH), 101.1 (d, *J* = 5.5 Hz, C_quat_), 86.5 (CH), 76.1 (C_quat_), 70.8 (CH_2_), 27.4 (CH), 19.0 (CH_3_). ^**19**^**F NMR** (375 MHz, d_6_-DMSO) δ = -121.8 (td, *J* = 9.4, 4.5 Hz). **LC-MS** Found : [M+H]^+^, 260.1.

#### Synthesis of compound AB668 isobutyl 5-fluoro-3-(1-((1-(2-((4-isobutylphenyl)sulfonamido)ethyl)piperidin-4-yl)methyl)-1*H*-1,2,3-triazol-4-yl)-1*H*-indole-2-carboxylate

In an oven-dried round bottom flask was placed under argon atmosphere the indole 7 (60 mg, 0.23 mmol, 1.00 equiv.) and azide 4 (92 mg, 0.24 mmol, 1.05 equiv.) in tetrahydrofuran - *tert*-butanol (579 μL, 2:1, C ∼ 0.4 M). A freshly prepared 2M aqueous of sodium ascorbate (405 μL, 0.81 mmol, 3.50 equiv.) and a 15% aqueous of copper(II) sulfate pentahydrate (337 μL, 0.20 mmol, 0.875 equiv.) were added through syringes and the reaction mixture was stirred vigorously at room temperature for 20 h until the complete consumption of the starting material (monitored by TLC). After completion, the mixture was poured into water and extracted with ethyl acetate three times (3 x 50 mL). The combined organic extracts were washed with brine (5à mL), dried (MgSO_4_) and filtered. The solvents were removed under reduced pressure and the residue was purified chromatographically on silica gel (eluting gradient dichloromethane - methanol 98:2 to 96:4) to provide isobutyl 5-fluoro-3-(1-((1-(2-((4-isobutylphenyl)sulfonamido)ethyl)piperidin-4-yl)methyl)-1*H*-1,2,3-triazol-4-yl)-1*H*-indole-2-carboxylate (AB668) as a white solid (91 mg, 62% yield).

#### AB668 isobutyl 5-fluoro-3-(1-((1-(2-((4-isobutylphenyl)sulfonamido)ethyl)piperidin-4-yl)methyl)-1*H*-1,2,3-triazol-4-yl)-1*H*-indole-2-carboxylate

**Mp** 161-163°C. ^**1**^**H NMR** (400 MHz, d_6_-DMSO) δ = 12.01 (s, 1H), 8.55 (s, 1H), 8.05 (dd, *J* = 10.2, 2.5 Hz, 1H), 7.70 (d, *J* = 8.3 Hz, 2H), 7.55 (dd, *J* = 9.0, 4.7 Hz, 1H), 7.34 (t, *J* = 8.4 Hz, 3H), 7.22 (td, *J* = 9.1, 2.6 Hz, 1H), 4.33 (d, *J* = 7.0 Hz, 2H), 4.12 (d, *J* = 6.7 Hz, 2H), 2.86-2.78 (m, 2H), 2.65 (d, *J* = 11.2 Hz, 2H), 2.48 (s, 2H), 2.23 (t, *J* = 6.8 Hz, 2H), 2.03 (dq, *J* = 13.4, 6.7 Hz, 1H), 1.81 (tt, *J* = 16.5, 9.0 Hz, 4H), 1.44 (d, *J* = 11.3 Hz, 2H), 1.25-1.16 (m, 2H), 0.94 (d, *J* = 6.7 Hz, 6H), 0.81 (d, *J* = 6.6 Hz, 6H). ^**13**^**C NMR** (100 MHz, d_6_-DMSO) δ = 161.0 (C_quat_), 157.5 (d, *J* = 234.1 Hz, C_quat_), 145.9 (C_quat_), 140.2 (C_quat_), 138.0 (C_quat_), 133.1 (C_quat_), 129.5 (CH), 126.4 (CH), 126.0 (d, *J* = 10.3 Hz, C_quat_), 124.7 (CH), 123.7 (C_quat_), 114.4 (d, *J* = 26.7 Hz, CH), 114.0 (d, *J* = 9.4 Hz, CH), 111.9 (d, *J* = 5.6 Hz, C_quat_), 107.3 (d, *J* = 24.6 Hz, CH), 70.6 (CH_2_), 56.8 (CH_2_), 54.4 (CH_2_), 52.5 (CH_2_), 44.1 (CH_2_), 40.2 (CH_2_), 36.5 (CH_2_), 29.5 (CH), 29.0 (CH_2_), 27.4 (CH), 22.0 (CH_3_), 18.9 (CH_3_). ^**19**^**F NMR** (376 MHz, d_6_-DMSO) δ = -122.4 (td, *J* = 9.7, 4.7 Hz). **HRMS (ESI**^**+**^**) :** calcd. for C_33_H_44_FN_6_O_4_S, 639.3122. Found : [M+H]^+^, 639.3123 (0.2 ppm error).

#### Recombinant proteins for enzymatic measurements

Both human recombinant CK2α subunit and chicken recombinant MBP (maltose-binding protein)-CK2β were expressed in *Escherichia coli* and purified as previously reported (Hériché et al., 1997; Chantalat et al., 1999). Proteins were quantified using a Bradford assay and the quality of the purification was asserted by SDS-PAGE analysis.

#### CK2 activity assays *in vitro*

Radiometric kinase assays were performed as previously reported (Kufareva et al., 2019). Briefly, in a final volume of 20 μL at 4°C, 3.0 μL of CK2α protein (36 ng) was incubated in the reaction mixture (20 mM Tris-HCl, pH 7.5, 150 mM NaCl, 1.0 mM DTT) with 1.0 mM of the synthetic substrate peptide, 20 mM of MgCl_2_, 1.0 μCi of [^32^P]-ATP and 2.0 μL of different concentrations of the inhibitor, diluted in Tris-HCl-glycerol, 0.05% Tween 20. Final ATP concentration was 10 μM when not stated otherwise. The kinase reactions were performed under linear kinetic conditions for 5 min at room temperature followed by quenching with the addition of 60 μL of 4% TCA. ^32^P incorporation in peptide substrate was determined by spotting the supernatant onto phospho-cellulose paper disks (Whatman P81, 4 cm^2^). The disks were washed three times in cold 0.5% phosphoric acid, 5 min on a rocking platform per wash, dried and finally the radioactivity was measured. Percentage inhibition was calculated relative to a DMSO control, and all measurements were performed in duplicate. A canonical CK2 peptide substrate (Seq. RRREDEESDDE) phosphorylated equally by CK2α_2_β_2_ (CK2β-independent) and a 22-residue long *N*-terminal fragment of the eukaryotic translation initiation factor 2 (eIF2) (.MSGDEMIFDPTMSKKKKKKKKP), exclusively phosphorylated by CK2α_2_β_2_ (CK2β-dependent) were used for the radiometric kinase assays. Phosphorylation assay using GST-SIX1 (3.7 μg) were performed in the same buffer. Final concentration of ATP was 100 μM. Samples were analyzed by SDS-PAGE and subjected to autoradiography. Phosphoproteins were quantified by densitometry scanning using ImageJ (National Institutes of Health software v1.52).

#### X-ray crystallography

Recombinant protein for X-ray studies was produced as published in Wells et al. (2021). Protein concentration was 9 mg/ml. Crystals of human CK2α were grown at 20°C using the hanging-drop vapor-diffusion method with a reservoir solution containing 33% polyethylene glycol methyl ether 5000, 0.2 M ammonium sulfate, 0.1 MES pH 6.5. The drops contained 1μl of the reservoir solution and 1 μl of the protein. The crystals were soaked by adding 0.2 μl of ligand solution at 100 mM in DMSO. The crystals were cryo-protected with reservoir solution supplemented with 20% glycerol and then flash-cooled in liquid nitrogen. X-ray diffraction data were collected at the ESRF Synchrotron in Grenoble, France, on beamline IB30B. Data were integrated and processed using XDS.^83^ The crystals belong to the space group P43212 with two monomers in the asymmetric unit. The structures were solved by molecular replacement using PDB entry 6Z84 as the search model. Bound ligands were manually identified and fitted into Fo–Fc electron density using Coot (Emsley & Cowtan, 2004). Files CIF format for ligand were generated using Grade Server (http://grade.globalphasing.org/cgi-bin/grade/server.cgi). The structure was refined by rounds of rebuilding in Coot and refinement using Phenix (Adams et al., 2010). Data collection and refinement statistics for crystal structure is presented in Table S1 and the PDB report is available in supplemental information.

#### Thermal shift assay

The thermal shift assay was performed on a LightCycler 480 Real-Time PCR System (Roche) in 96-well white plates (Armadillo plate, Thermo Scientific) using an integration time of 120ms. Each well contained 10 μL of 5μg CK2α (purified as described by Hériché et al., 1997; Chantalat et al., 1999) and 2.5× SYPRO Orange (Life Technologies) in PBS-0.9% glycerol, with ligands added to a final concentration of 0.1μM to 500μM in 5% (v/v) DMSO. All assays were carried out in triplicate. Each plate was sealed with an optically clear foil and centrifuged for 1 min at 300 rpm before performing the assay. The plates were heated from 20 to 80°C at a heating rate 0.01°C/s. The fluorescence intensity was measured with λex = 483 nm and λem = 568 nm. The melting temperature (Tm) was determined using the TSA-CRAFT software that enables automatic analysis of TSA data exported from the Roche Lightcycler 480 software (Lee et al., 2019).

#### Kinase screening

Profiling of 468 recombinant protein kinases was performed by Eurofins Discovery (KINOMEscan™ Profiling Service, San Diego, USA) in the presence of 2 μM AB668, using an active site-directed competition binding assay. In this assay, an ATP-site kinase ligand is immobilized. The DNA-tagged kinases are captured in the absence and the presence of AB668. Competition is measured using a qPCR method that detects the associated DNA label. In addition, dissociation constants were measured using the same assay for the kinases MARK3 and PIKFYVE as the screening assay indicated a strong inhibition comparable to CK2a and CK2a’. Further information are shown in the KINOMEscan™ Profiling report).

#### Caspase-3 assay

The Caspase-3 Fluorometric Assay Kit II from Biovision was used to determine caspase-3 activity in cells following manufacturer’s instructions. Briefly, cultured cells collected by scraping were lyzed and Bradford reagent was used to quantify proteins in the cell lysate. Fifty micrograms of proteins were added in a 96-well white plate. Reaction was started by adding the caspase-3 substrate, DEVD-AFC. Fluorescence measurements (excitation: 405 nm; emission: 520 nm) were made at 37°C in a FLUOstar OPTIMA microplate reader (BMG Labtech) every 5 min over an hour. Cells treated with 500 nM staurosporine, a potent apoptotis inducer, were used as positive controls.

#### Cell death & proliferation

786-O (2 x 10^4^ cells per well), HEK293 (2 x 10^4^ cells per well), MCF10A (2 x 10^4^ cells per well), MDA-MB231 (2 x 10^4^ cells per well) and RPTEC (7 x 10^3^ cells per well) cells were seeded into 96-well flat-bottom cell culture plates. After 24 h, compounds dissolved in DMSO were diluted in the culture medium containing 0.5 μg/mL propidium iodide (PI, Sigma-Aldrich) and were added to the cell culture media such as the final DMSO concentrations is equal to 0.2% (v/v). Experiments were conducted at 37°C in a 5% CO_2_ atmosphere and the plates were tracked using an Essen IncuCyte Zoom live-cell microscopy instrument. For cell death, PI-stained red fluorescent cells images were captured every 3 h for the entire duration of the experiment and normalized to the DMSO standard control. For cell proliferation, the software incorporated into the IncuCyte Zoom was specifically calibrated to ensure accurate distinction of cells from the empty spaces. Cell proliferation was monitored by analyzing the occupied area (% confluence) of cell images over time.

#### Real-time proliferation, cell death and apoptosis assay on A375 cells

A375 melanoma cells (2.5 x 10^4^ cells per well) were plated in 48-well plates. After 24 hours, cells were treated with various doses of AB668, CX4945, or SGC-CK2-1. Treatment medium contained 0.3 μg/mL propidium iodide (Sigma-Aldrich) and 2 μM CellEvent™ Caspase-3/7 Green Detection Reagent (ThermoFisher Scientific). Images were captured automatically every two hours for 48 hours using the IncuCyte™ S3 Live-Cell Analysis Instrument (Essen BioScience). Image analysis was performed using IncuCyte software. Data was plotted as mean ± SD using GraphPad Prism 9.

#### Preparation of cell extracts

786-O (3 x 10^5^ cells per well), HEK 293 (2 x 10^5^ cells per well), MDA-MB231 (3 x 10^5^ cells per well), and RPTEC (3 x 10^5^ cells per well) cells were seeded into 6-well tissue and cultured for 24 h prior to the addition of inhibitors (as described above). After incubation, medium was removed, cells were washed with cold PBS and frozen at -80°C. Cells were lysed using a RIPA buffer (10 mM Tris-HCl, pH 7.4, 150 mM NaCl, 1% Triton X-100, 0.1% SDS, 0.5% DOC and 1.0 mM EDTA) with the addition of protease and phosphatase inhibitor cocktail (Sigma-Aldrich, P8340, P2850, P5726) at the recommended concentrations. Cell pellets were incubated in RIPA buffer on ice for 30 min, then centrifuged for 15 min at 4°C at 13000 rpm and the supernatants collected. Proteins were quantified using the Pierce BCA protein assay kit (Pierce, ThermoFisher Scientific).

#### CK2 activity in cells

Cell homogenates were assayed for CK2 activity with radiometric assays as described above.

#### Immunoblotting

Equal amounts of lysates (20-35 μg) were loaded onto a precast 4-12% gradient gel (Bio-Rad) and submitted to electrophoresis in NuPAGE buffer (150 V for 1.5 h). The gels were transferred onto PVDF membrane (100 V for 1 h). Membranes were blocked during 1 h at room temperature with saturation buffer (5% BSA in Tris-buffered saline with 0.1% Tween 20 (TBST) and then incubated with primary antibody diluted in saturation buffer for 2 h or overnight on a rocking platform shaker. Primary antibodies were GAPDH antibody (#AM4300) from Invitrogen, ThermoFisher, P-AKT-phospho-Ser129 (#AP3020a) from Interchim, AKT (#9272), PARP (#9542), mTOR (#2972), mTOR-phospho-Ser2448 (#2971), p38MAPK (#9212), p38MAPK-phospho-Thr180/Tyr182 (#9211), p53 (#9282), p53-phospho-Ser15 (#9286), STAT3 (#6139), STAT3-phospho-Ser727 (#9134) antibodies from Cell Signaling, survivin (#NB500201) antibody from Novus biologicals, p21 antibody (#sc-397) from Santa Cruz Biotechnologies, p21-phospho-Thr145(#ab-47300) antibody from Abcam. After washing three times with TBST, secondary antibody (peroxidase-conjugated affinity pure Goat anti-rabbit IgG (#111035003) or peroxidase-conjugated affinity pure goat anti-mouse IgG (#115035003) from Jackson Immuno Research) was added for 1 h followed by three more washes with TBST. Immobilon Forte Western HRP substrate (Millipore) was added and detection was achieved by using Fusion FX acquisition system (Vilbert). Anti-GAPDH was used as loading control and images were analyzed and band intensities were quantified using ImageJ (National Institutes of Health software v1.52).

#### Fresh tissue sectioning

A Vibratome VT1200 (Leica Microsystems) was used to cut thin (300 μm) slices from fresh tissue. Samples were soaked in ice-cold sterile balanced salt solution (HBSS), orientated, mounted, and immobilized using cyanoacrylate glue. Slicing speed was optimized according to tissue density and type; in general, slower slicing speed was used on the softer tissues and vice versa (0.08-0.12 mm/s neoplastic tissue; 0.01-0.08 mm/s normal tissue). Vibration amplitude was set at 2.95-3.0 mm.

#### Organotypic tissue cultures

Tissue slices were cultured on organotypic inserts for 48 h (one slice per insert; Millipore). Organotypic inserts are Teflon membranes with 0.4 μm pores that allow preservation of 3D tissue structure in culture. Tissue culture was performed at 37°C in a 5 % CO_2_ humidified incubator using 1 ml of DMEM media supplemented with 20 % FBS (GIBCO), 100 U/ml penicillin (Invitrogen) and place in a rotor agitator to allow gas and fluids exchanges with the medium. The tissue slices were incubated with the CX-4945 (10 μM) or AB668 (5 μM) until 68 hours. Tumor slice viability was monitored at indicated time points, by luminescence imaging of 786-O-luc cells after luciferin addition (IVIS) as previously described (Roelants et al., 2018; Roelants et al., 2020).^47^^,^^48^

#### BRB-seq library preparation and sequencing

786-O cells (150, 000) were treated for 24h with DMSO, 5 μM of CX4945 or AB668. Total RNA was extracted from treated cells using the MirVana PARIS kit (Thermofisher). The 3′ Bulk RNA Barcoding and sequencing (BRB-seq) experiments were performed at the Research Institute for Environmental and Occupational Health (Irset, Rennes, France) according to the published protocol.^84^ Briefly, the reverse transcription and the template switching reactions were performed using 4 μl total RNA at 2.5 ng/μl. RNA were first mixed with 1 μl barcoded oligo-dT (10 μM BU3 primers, Microsynth), 1 μL dNTP (0.2 mM) in a PCR plate, incubated at 65°C for 5 min and then put on ice. The first-strand synthesis reactions were performed in 10 μl total volume with 5 μl of RT Buffer and 0.125 μl of Maxima H minus Reverse Transcriptase (Thermofisher Scientific, #EP0753) and 1 μl of 10 μM template switch oligo (TSO, IDT). The plates were then incubated at 42°C for 90 min and then put on ice.

After Reverse Transcription (RT), decorated cDNA from multiple samples were pooled together and purified using the DNA Clean & concentrator-5 Kit (Zymo research, #D4014). After elution with 20 μl of nuclease free water, the samples were incubated with 1 μl Exonuclease I (NEB, #M0293) and 2 μl of 10X reaction buffer at 37°C for 30 min, followed by enzyme inactivation at 80°C for 20 min.

Double-strand (ds) cDNAs were generated by PCR amplification in 50 μl-total reaction volume using the Advantage 2 PCR Enzyme System (Clontech, #639206). PCR reaction was performed using 20 μl cDNA from previous step, 5 μl of 10X Advantage 2 PCR buffer, 1 μl of dNTPs 50X, 1 μl of 10 μM LA-oligo (Microsynt), 1 μl of Advantage 2 Polymerase and 22 μl of nuclease-free water following the program (95°C-1 min; 11 cycles: 95°C-15 s, 65°C-30 s, 68°C-6 min; 72°C-10 min). Full-length double-stranded cDNA was purified with 30 μl of AMPure XP magnetic beads (Beckman Coulter, #A63881), eluted in 12 μl of nuclease free water and quantify using the dsDNA QuantiFluor Dye System (Promega, #E2670).

The sequencing libraries were built by tagmentation using 50 ng of ds cDNA with Illumina Nextera XT Kit (Illumina, #FC-131-1024) following the manufacturer’s recommendations. The reaction was incubated 5 min at 55°C, immediately purified with DNA Clean & concentrator-5 Kit (Zymo research) and eluted with 21 μl of nuclease free water. Tagmented library was PCR amplified using 20 μl eluted cDNA, 2.5 μl of i7 Illumina Index, 2.5 μl of 5 μM P5-BRB primer (IDT) using the following program (72°C-3 min; 98°C-30 s; 13 cycles: 98°C-10 s, 63°C-30 s, 72°C-5 min). The fragments ranging 300-800 base pair (bp) were size-selected using SPRIselect (Bekman Coulter, #) (first round 0.65x beads, second 0.56x) with a final elution of 12 μl nuclease-free water. The resulting library was sequenced on Illumina Hiseq 4000 sequencer as Paired-End 100 base reads following Illumina’s instructions. Image analysis and base calling were performed using RTA 2.7.7 and bcl2fastq 2.17.1.14. Adapter dimer reads were removed using DimerRemover (https://sourceforge.net/projects/dimerremover/). Data are available at https://www.ebi.ac.uk/biostudies/arrayexpress/studies/E-MTAB-12776.

#### BRB-seq raw data preprocessing

The first read contains 16 bases that must have a quality score higher than 10. The first 6 bp correspond to a unique sample-specific barcode and the following 10 bp to a unique molecular identifier (UMI). The second reads were aligned to the human reference transcriptome from the UCSC (University of California, Santa Cruz) website (release hg38) using BWA version 0.7.4.4 with the non-default parameter “−l 24”. Reads mapping to several positions in the genome were filtered out from the analysis. The pipeline has been previsouly described.^85^ After quality control and data preprocessing, a gene count matrix was generated by counting the number of unique UMIs associated with each gene (lines) for each sample (columns). The resulting UMI matrix was further normalized by using the rlog transformation implemented in the DeSeq2 package.^86^

#### Bioinformatics analysis

Differential Gene Expression (DGE) analyses were performed using DeSeq2 R package (version 1.34.0, R 4.1) for each pairwise comparison of AB668, CX-4945 and DMSO conditions (Table S2). For these 3 DGE results, p-values were corrected by the Benjamini-Hochberg method. Only genes with corrected P-values < 0.05 and |Log2(Fold Change)|> 0.5 were considered as significantly differentially expressed. The first sample label in a comparison (e.g. AB668 for "AB668 vs DMSO") means that it is the numerator in the calculation of the fold change (Fold Change = AB668/DMSO). The top 15 of significant genes based on FDR values and apoptotic and lipid & steroid metabolism related genes (if not overlapping with the top 15) are displayed in volcano plots. All DGE values for each selected genes in three comparisons can be retrieved in the supp-data (volcanoPlots3_AB668_CX_DMSO.ods). Gene Set Enrichment Analysis (GSEA) of Reactome pathway database (reactome.db version 1.82.0) was carried out using the Bioconductor/R package clusterProfiler (version 4.6.0). Pathways are considered significantly enriched whether related corrected P-value (Benjamini-Hochberg correction) is lower than 0.05.

### Quantification and statistical analysis

Statistical analyses were performed using GraphPad Prism 8 and all the statistical details of experiments can be found in Figure legends. Data were expressed as mean ± standard error (mean + SEM) unless otherwise stated. Measurement data with normal distribution were tested by independent sample t-test. Kruskal-Wallis one-way ANOVA was used for non-normally distributed data. P-values less than 0.05 were considered statistically significant (∗p < 0.05, ∗∗p < 0.01, ∗∗∗p < 0.001, ∗∗∗∗p < 0.0001).

For bioinformatic analysis, statistical analyses were performed using R software (v4.1). The selection of significantly differentially expressed genes, between pairwise comparisons of sample groups, was performed with the Wald statistic. The selection of biological pathways significantly over-represented in deregulated genes was carried out using the weighted Kolmogorov–Smirnov statistic. Adjustment of P-values to take into account multiple testing was done with the Benjamini-Hochberg correction. P-values less than 0.05 were considered statistically significant unless otherwise stated.

## Supplemental information

Document S1. Figures S1–S12 and Table S1

Data S1. Kinase scan for AB668, performed using KINOME*scan*™ screening platform, related to Figure 1

Table S2. BRB-seq data, related to Figure 4
