# Supplementary material for: Cancer selective cell death induction by a bivalent CK2 inhibitor targeting the ATP site and the allosteric αD pocket
Source: iScience. 2024 Jan 12;27(2):108903. doi: 10.1016/j.isci.2024.108903 (PMC10838953; doi:10.1016/j.isci.2024.108903)
Supplement: Document S1. Figures S1–S12 and Table S1 [file mmc1.pdf]

iScience, Volume *27*

Supplemental information

Cancer selective cell death induction by a bivalent CK2 inhibitor targeting the ATP site and the allosteric αD pocket

Alexandre Bancet, Rita Frem, Florian Jeanneret, Angélique Mularoni, Pauline Bazelle, Caroline Roelants, Jean-Guy Delcros, Jean-François Guichou, Catherine Pillet, Isabelle Coste, Toufic Renno, Christophe Battail, Claude Cochet, Thierry Lomberget, Odile Filhol, and Isabelle Krimm

Figure S1. Binding sites and chemical structures of small-molecule CK2 inhibitors, related to Figure 1. Compound 4 is a CK2 inhibitor that binds at the CK2α/CK2b interface; CX-4945 and SGC-CK2-1 are ATP-competitive inhibitors; CAM4066 and KN2 are bivalent inhibitors that bind at the ATP site and in the αD pocket of CK2. References and PDB codes are indicated for each molecule.

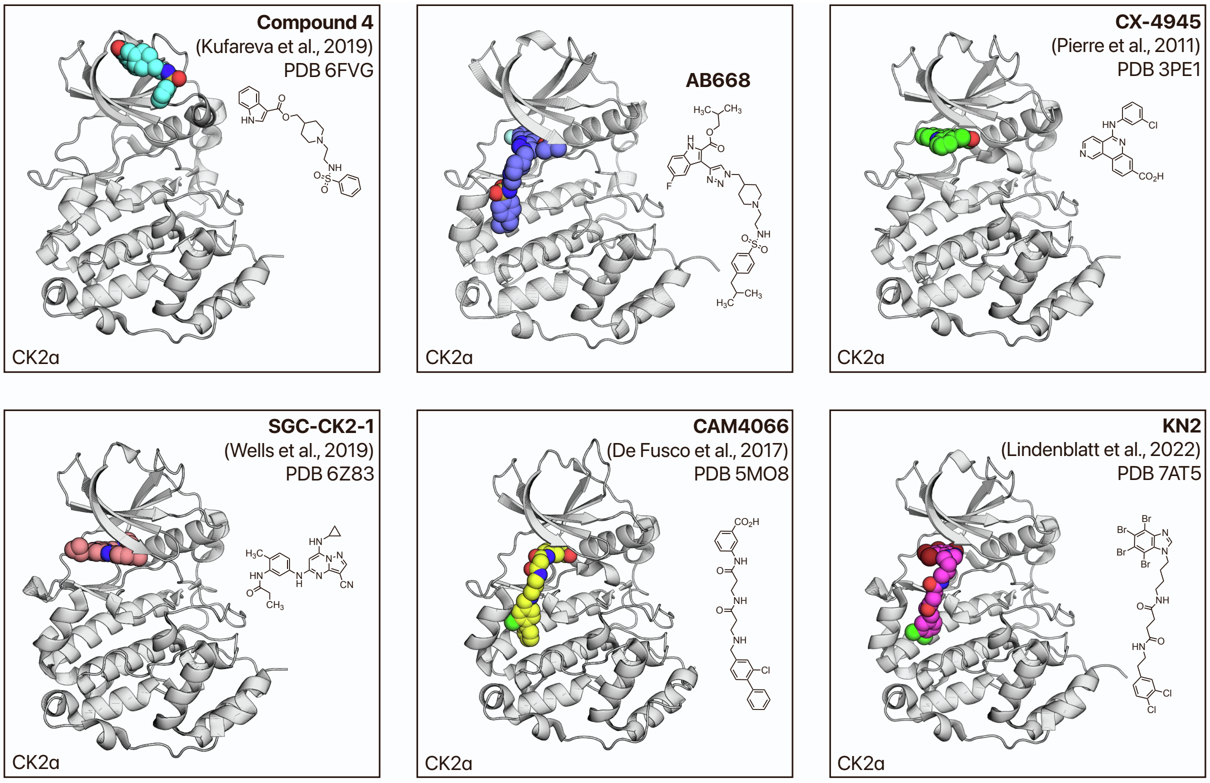

Figure S2. OMIT electron-density-map for AB668 bound to CK2α, related to Figure 1. The purple mesh and surface represent the electron density map ($2F_O–F_C$ omit map) contoured at 1.0 σ of AB668 bound to CK2α.

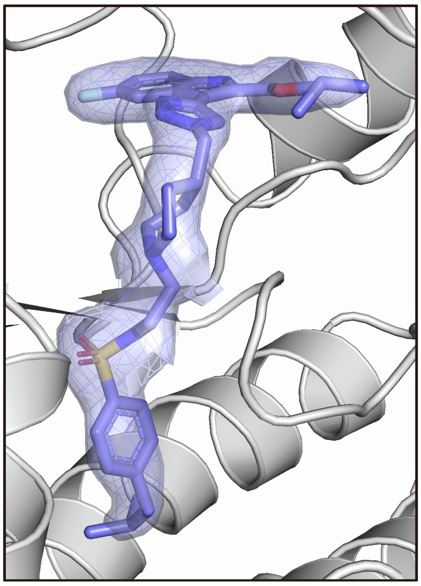

Figure S3. ^{1}H NMR spectrum of AB668, related to Figure 1. ^{1}H NMR (400 MHz, d_6-DMSO) δ = 12.01 (, related to Figure 1s, 1H), 8.55 (s, 1H), 8.05 (dd, *J* = 10.2, 2.5 Hz, 1H), 7.70 (d, *J* = 8.3 Hz, 2H), 7.55 (dd, *J* = 9.0, 4.7 Hz, 1H), 7.34 (t, *J* = 8.4 Hz, 3H), 7.22 (td, *J* = 9.1, 2.6 Hz, 1H), 4.33 (d, *J* = 7.0 Hz, 2H), 4.12 (d, *J* = 6.7 Hz, 2H), 2.86-2.78 (m, 2H), 2.65 (d, *J* = 11.2 Hz, 2H), 2.48 (s, 2H), 2.23 (t, *J* = 6.8 Hz, 2H), 2.03 (dq, *J* = 13.4, 6.7 Hz, 1H), 1.81 (tt, *J* = 16.5, 9.0 Hz, 4H), 1.44 (d, *J* = 11.3 Hz, 2H), 1.25-1.16 (m, 2H), 0.94 (d, *J* = 6.7 Hz, 6H), 0.81 (d, *J* = 6.6 Hz, 6H).

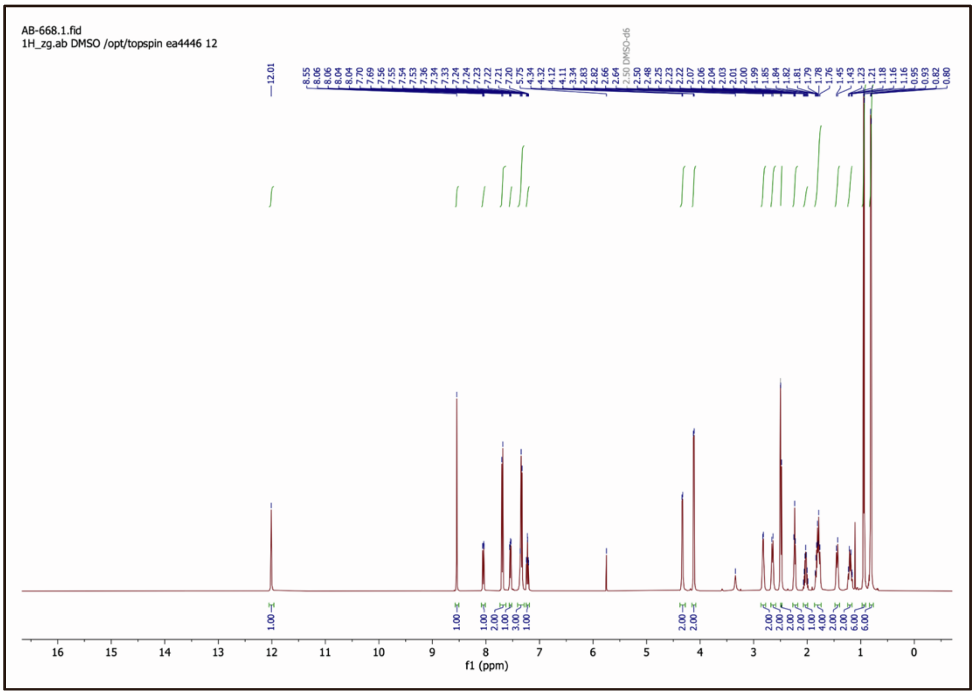

Figure S4. ^{13}C NMR spectrum of AB668, related to Figure 1. ^{13}C NMR (100 MHz, d_6-DMSO) δ = 161.0 (C_{quat}), 157.5 (d, *J* = 234.1 Hz, C_{quat}), 145.9 (C_{quat}), 140.2 (C_{quat}), 138.0 (C_{quat}), 133.1 (C_{quat}), 129.5 (CH), 126.4 (CH), 126.0 (d, *J* = 10.3 Hz, C_{quat}), 124.7 (CH), 123.7 (C_{quat}), 114.4 (d, *J* = 26.7 Hz, CH), 114.0 (d, *J* = 9.4 Hz, CH), 111.9 (d, *J* = 5.6 Hz, C_{quat}), 107.3 (d, *J* = 24.6 Hz, CH), 70.6 (CH_2), 56.8 (CH_2), 54.4 (CH_2), 52.5 (CH_2), 44.1 (CH_2), 40.2 (CH_2), 36.5 (CH_2), 29.5 (CH), 29.0 (CH_2), 27.4 (CH), 22.0 (CH_3), 18.9 (CH_3).

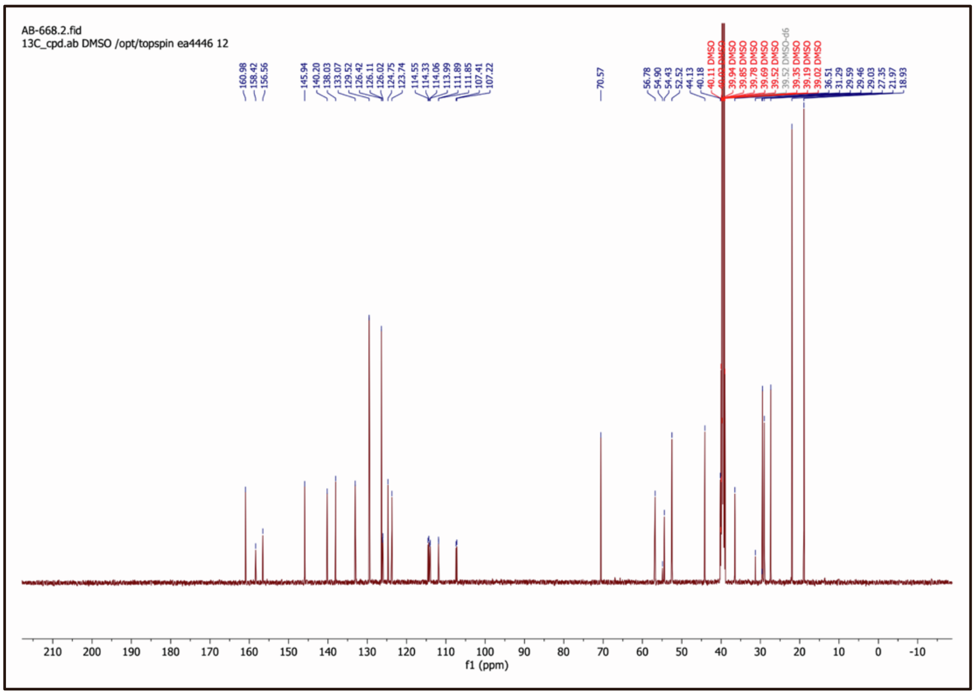

Figure S5. ^{19}F NMR spectrum of AB668, related to Figure 1. ^{19}F NMR (376 MHz, d_6-DMSO) δ = -122.4 (td, J = 9.7, 4.7 Hz).

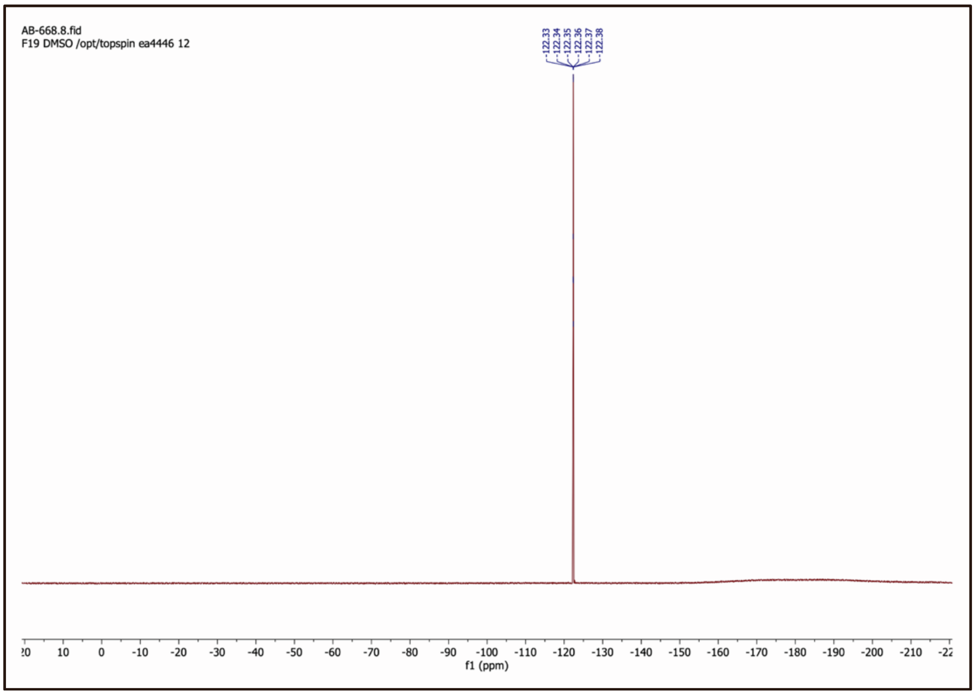

Figure S6. Thermal Shift and radiometric Assay experiments for the bivalent CK2 inhibitor AB668 and the ATP-competitive inhibitors CX-4945 and SGC-CK2-1 bound to CK2α, related to Figure1. **a**) Thermal Shift Assay melting curves observed for CK2 alone (blue) and in the presence of the three inhibitors (25 mM). CK2α alone displayed a Tm of 43.6 ± 0.4°C. **b**) Variation of CK2α ΔTm upon inhibitor addition is reported as a function of the ligand concentration. At saturating inhibitor concentration, CX-4945 induced a ΔTm = 13.8 ± 0.4°C, SGC-CK2-1 a ΔTm = 11.0 ± 0.3°C and AB668 a ΔTm = 5.2 ± 0.4°C. **c**) Radiometric phosphorylation assay of CK2 in the presence of increasing concentrations of the three inhibitors. Ki was determined as $IC_{50}/2$.

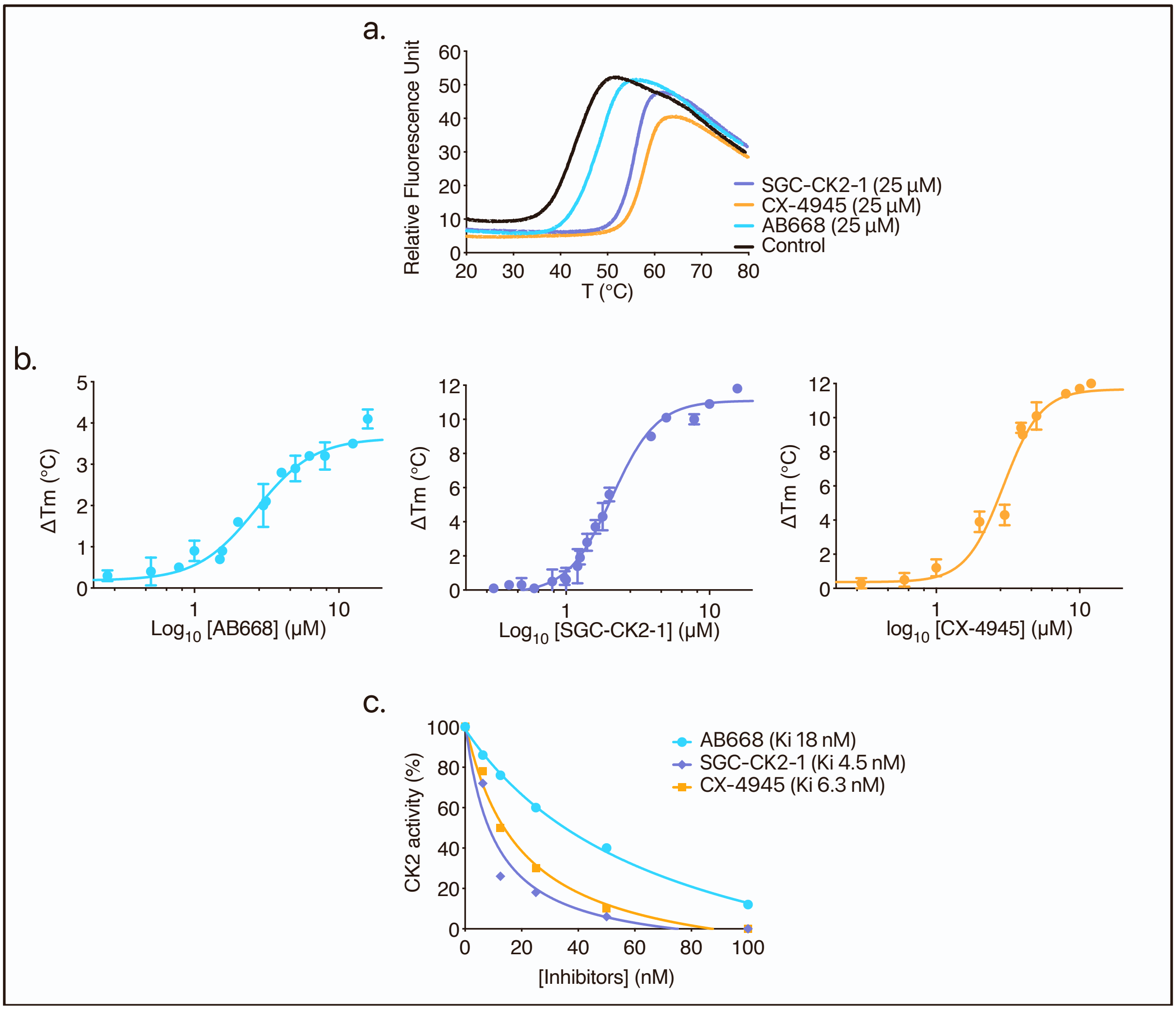

Figure S7. Inhibition of CK2 by AB668, related to Figure1. a) Inhibition constant for CK2 holoenzyme as determined using a canonical radiometric assay using a CK2β-dependent peptide substrate. **b)** Inhibition of SIX1 by AB668. AB668 inhibits the phosphorylation of SIX1, a transcription factor that is phosphorylated by the holoenzyme. At 500 nM, AB668 inhibits about 90% of SIX1 phosphorylation.

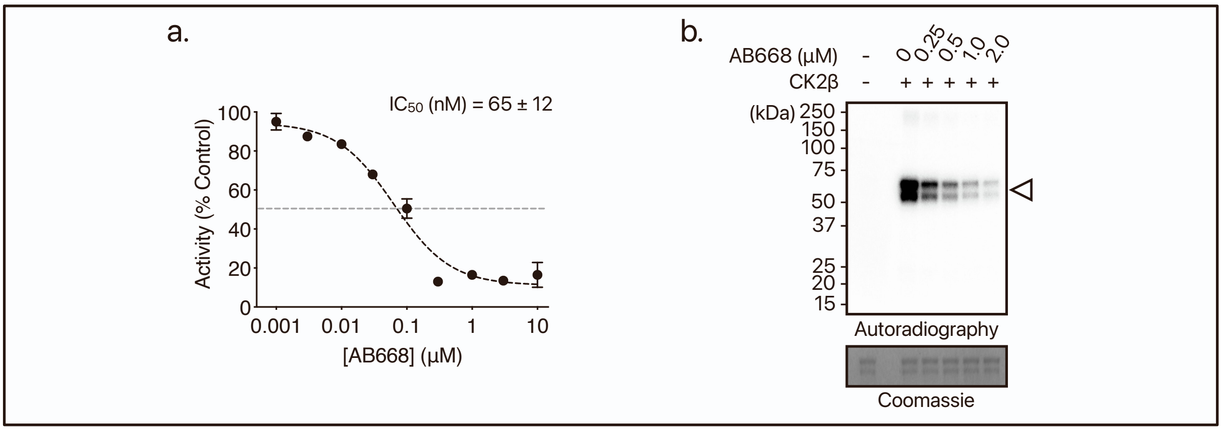

Figure S8. Effect of AB668 on catalytic activity of recombinant CK2α and CK2α', related to Figure 1. CK2 activity was measured by radiometric assay using 36 ng of CK2α (●) or CK2α' (☐) recombinant catalytic subunits.

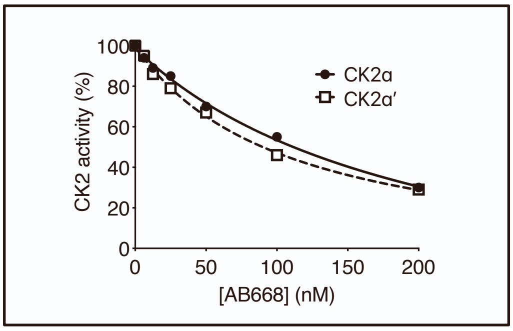

Figure S9. Cell mortality in 786-O cells in response to AB668, SGC-CK2-1 or CX-4945, related to Figure 2. Live cell imaging showing cell death in 786-O cells treated with increasing concentrations of AB668, CX-4945 or SGC-CK2-1 for 48 h.

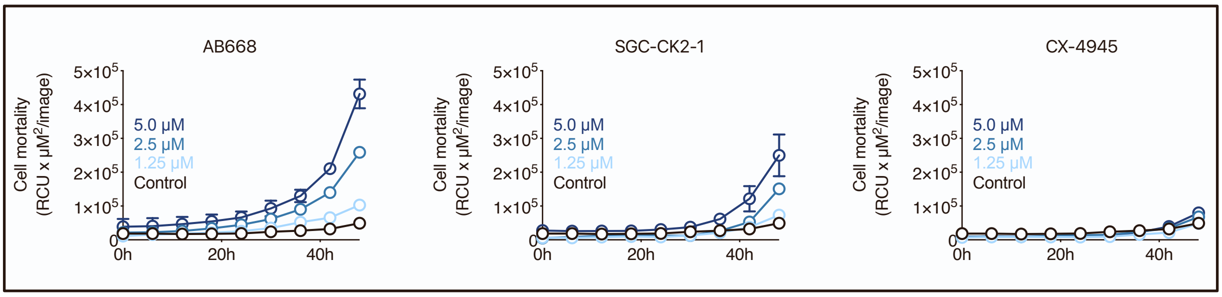

Figure S10. Cell mortality in 786-O, HEK293 or RPTEC cells treated with AB668, related to Figure 2. Live cell imaging showing cell death in 786-O, RPTEC or HEK293 cells treated with increasing concentrations of AB668, CX-4945 or SGC-CK2-1 for 72 h.

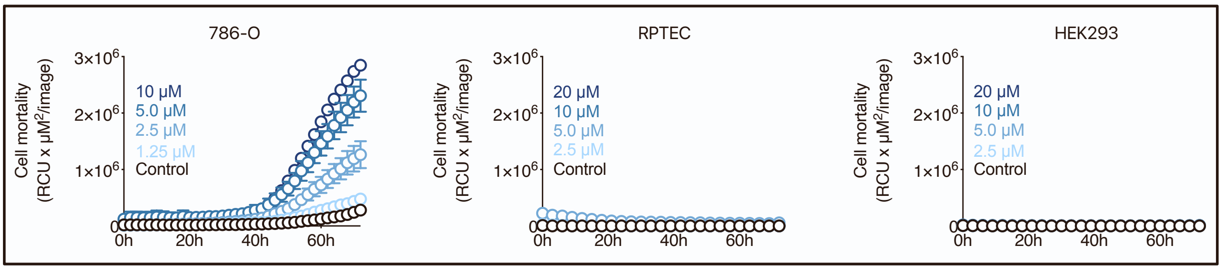

Figure S11. Impact of AB668 in cancer cells, related to Figures 2 and 3. a) Live cell imaging showing cell death in various cancer cell models (786-O, A549, MDA-MB-231, OVCAR-3, U373 MG) and normal MCF10A cells treated with increasing concentrations of AB668 ; **b**) Western blot analysis from the corresponding MDA-MB-231 cell extracts after 48 h of treatment showing the cleavage of PARP as well as the expression level of survivin. Phosphorylated STAT3 (S727), AKT (S129), p53 (S15), p38 MAPK (T180/Y182) and p21 (T145) were analyzed. Glyceraldehyde-3-phosphate dehydrogenase (GAPDH) was used as a loading control.

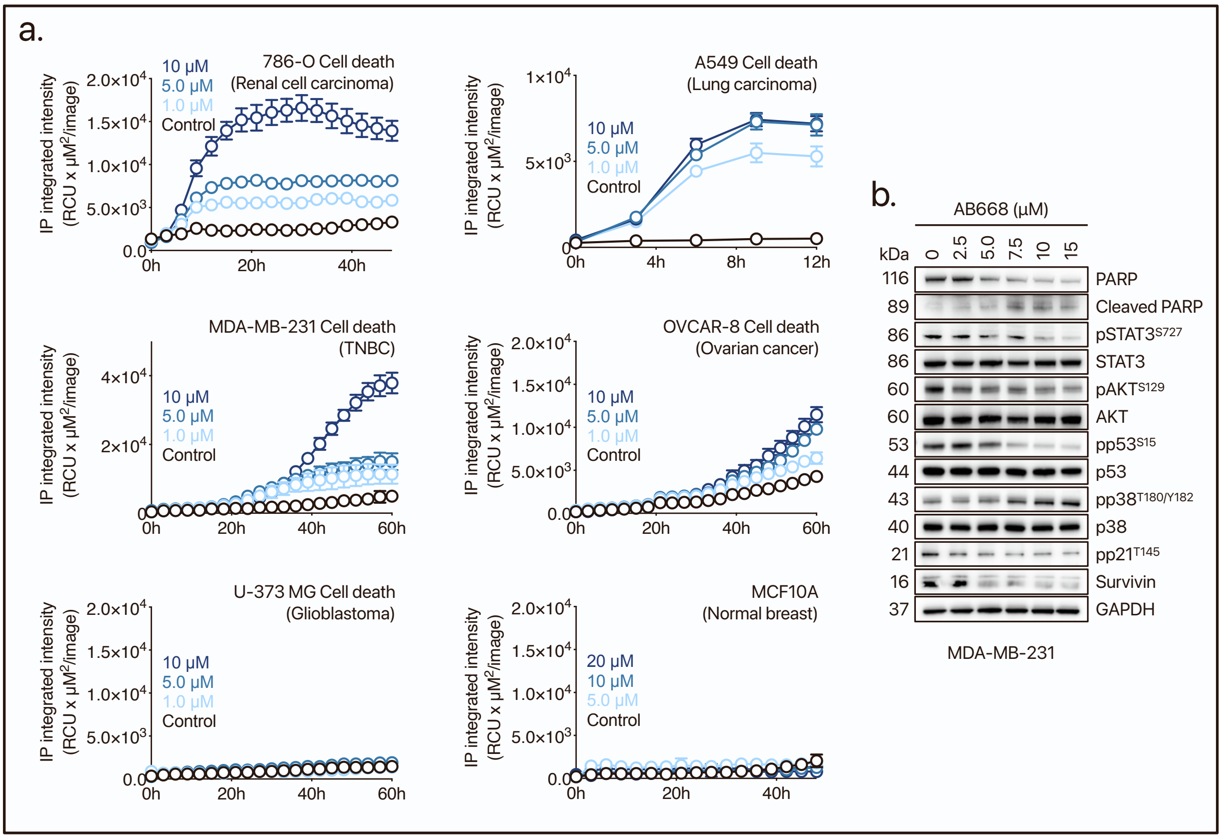

Figure S12. Persistence of cellular effects of CK2 inhibitors, related to Figures 2 and 3. 786-O cells were treated for 12 h with 5 μM of AB668, SGC-CK2, CX-4945 or 0.1 % DMSO. Then, cells were washed with PBS and immediately lysed (time 0) or further cultured for 8 h without inhibitor in the medium (time 8). **a)** CK2 activity measured by radiometric kinase assay with the CK2 consensus peptide substrate. b) Corresponding cell extracts were also incubated with ^{32}P-ATP and phosphorylated proteins were separated by SDS-PAGE and auto-radiographed. The last lane correspond to control cell lysate that was incubated in the presence of an excess of CK2 consensus peptide substrate. **c)** The same cell extracts were analyzed by Western blot using a CK2 substrates antibody. Glyceraldehyde-3-phosphate dehydrogenase (GAPDH) was used as a loading control. **d)** Phosphorylated AKT(S129) was analysed by Western blot in the same cell extracts. AKT was used as a loading control.

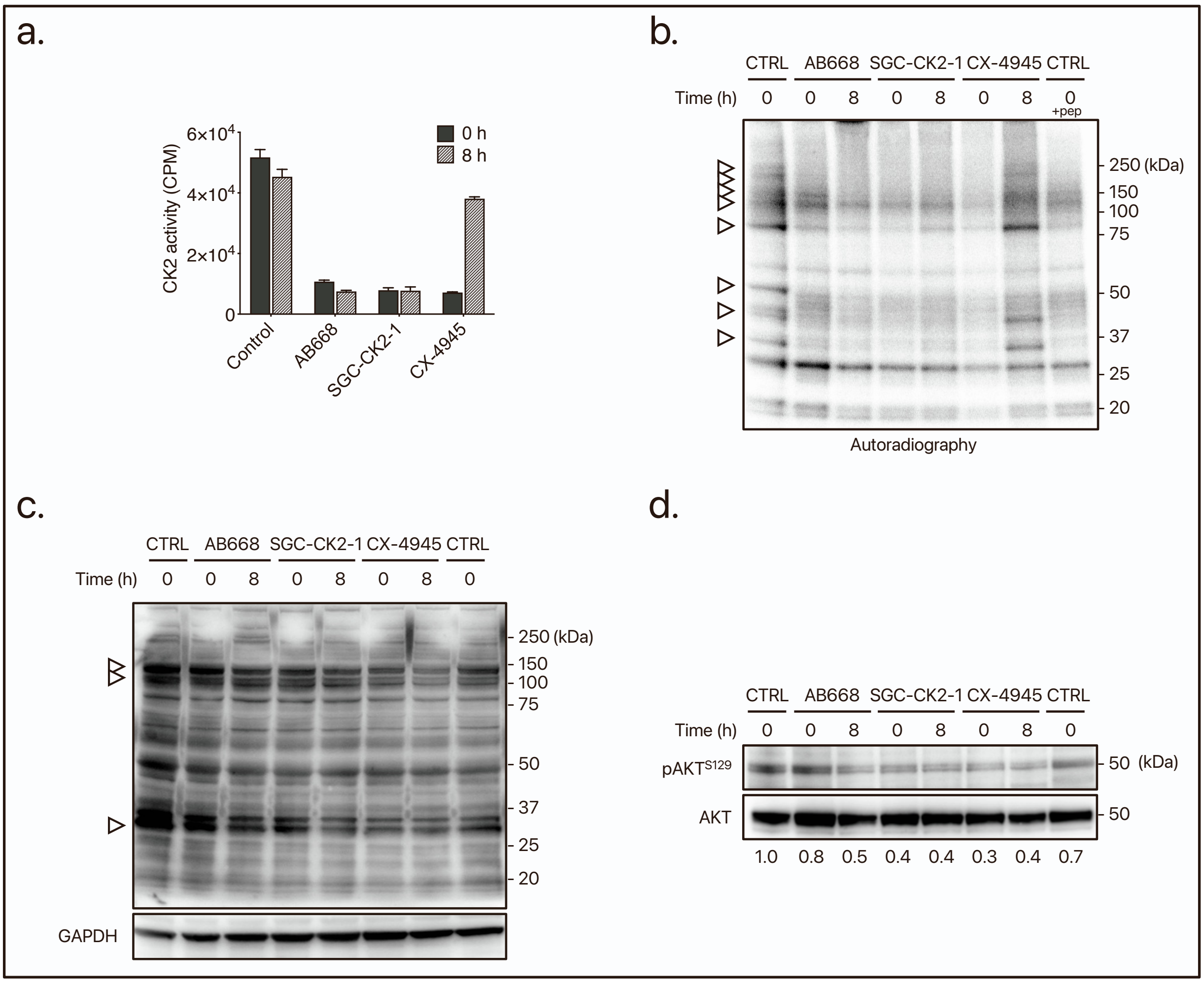

Table S1. Data collection and refinement statistics (molecular replacement), related to Figure 1.

	AB668
**Data collection**	
Wavelength (Å)	0.97625
Space group	$P43_12$
Cell dimensions	
a, b, c (Å)	127.521 127.521 125.065
α, β, γ (°)	90.0, 90.0, 90.0
Resolution (Å)	51.64-2.50 (2.59-2.50) *
Total reflections	200173 (19633)
Unique reflections	36228 (3561)
Multiplicity	5.5 (5.5)
Completeness (%)	99.7 (100)
Mean I / σI	11.5 (1.2)
Wilson B factor (Å^2)	61.25
R_{merge}	0.095 (0. 774)
Rmeas	0.106 (0.858)
Rp.i.m	0.045 (0.362)
$CC_{1/2}$	0.984 (0.672)
Completeness (%)	99.9 (100.0)
Redundancy	5.5 (5.5)
**Refinement**	
R_{work} / R_{free}	21.54/25.53
No. atoms	
Protein	5552
Ligand	207
Water	54
B-factors	
Protein	64.60
Ligand	71.33
Water	58.65
R.m.s. deviations	
Bond lengths (Å)	0.006
Bond angles (°)	0.766
Ramachadran allowed	6.10
Ramachandran favoured	93.14
No. TLS group	6

*Values in parentheses are for highest-resolution shell.
