# Supplementary material for: Cancer selective cell death induction by a bivalent CK2 inhibitor targeting the ATP site and the allosteric αD pocket
Source: iScience. 2024 Jan 12;27(2):108903. doi: 10.1016/j.isci.2024.108903 (PMC10838953; doi:10.1016/j.isci.2024.108903)

Data S1: Kinase scan for AB668, performed using KINOME*scan*™ screening platform, related to Figure 1.

Technology Overview

The KINOME*scan*™ screening platform employs a novel and proprietary active site-directed competition binding assay to quantitatively measure interactions between test compounds and more than 450 human kinases and disease relevant mutant variants. This robust and reliable assay technology affords investigators the ability to extensively annotate compounds with accurate, precise and reproducible data. KINOME*scan*™ assays do not require ATP and thereby report true thermodynamic interaction affinities, as opposed to IC50 values, which can depend on the ATP concentration.

How KINOME*scan*™ Works

Compounds that bind the kinase active site and directly (sterically) or indirectly (allosterically) prevent kinase binding to the immobilized ligand, will reduce the amount of kinase captured on the solid support (A & B). Conversely, test molecules that do not bind the kinase have no effect on the amount of kinase captured on the solid support (C). Screening "hits" are identified by measuring the amount of kinase captured in test versus control samples by using a quantitative, precise and ultra-sensitive qPCR method that detects the associated DNA label (D). In a similar manner, dissociation constants (Kds) for test compound-kinase interactions are calculated by measuring the amount of kinase captured on the solid support as a function of the test compound concentration.

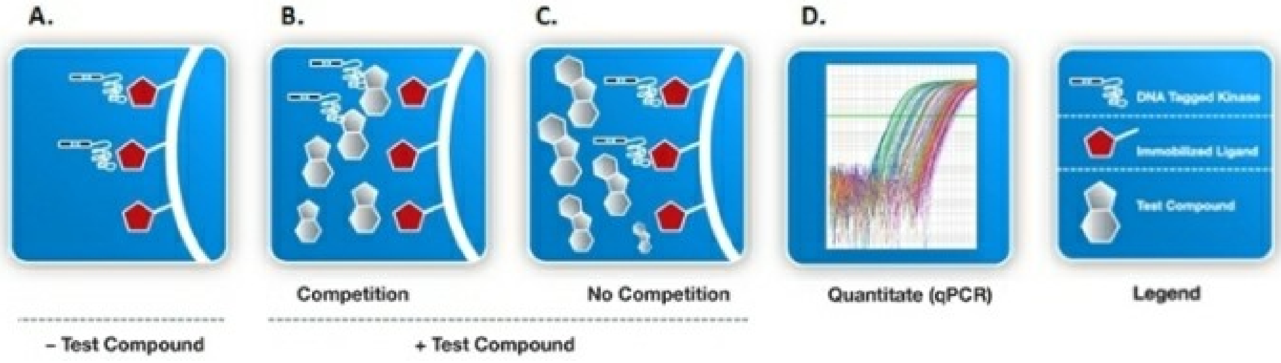

Protocol Description

Kinase assays. For most assays, kinase-tagged T7 phage strains were grown in parallel in 24-well blocks in an *E. coli* host derived from the BL21 strain. *E. coli* were grown to log-phase and infected with T7 phage from a frozen stock (multiplicity of infection = 0.4) and incubated with shaking at 32°C until lysis (90-150 minutes). The lysates were centrifuged (6,000 x g) and filtered (0.2μm) to remove cell debris. The remaining kinases were produced in HEK-293 cells and subsequently tagged with DNA for qPCR detection. Streptavidin-coated magnetic beads were treated with biotinylated small molecule ligands for 30 minutes at room temperature to generate affinity resins for kinase assays. The liganded beads were blocked with excess biotin and washed with blocking buffer (SeaBlock (Pierce), 1 % BSA, 0.05 % Tween 20, 1 mM DTT) to remove unbound ligand and to reduce non-specific phage binding. Binding reactions were assembled by combining kinases, liganded affinity beads, and test compounds in 1x binding buffer (20 % SeaBlock, 0.17x PBS, 0.05 % Tween 20, 6 mM DTT). Test compounds were prepared as 40x stocks in 100% DMSO and directly diluted into the assay. All reactions were performed in polypropylene 384-well plates in a final volume of 0.02 ml. The assay plates were incubated at room temperature with shaking for 1 hour and the affinity beads were washed with wash buffer (1x PBS, 0.05 % Tween 20). The beads were then re-suspended in elution buffer (1x PBS, 0.05 % Tween 20, 0.5 μM non-biotinylated affinity ligand) and incubated at room temperature with shaking for 30 minutes. The kinase concentration in the eluates was measured by qPCR.

Percent Control (%Ctrl)

The compound(s) were screened at the concentration(s) requested, and results for primary screen binding interactions are reported as '% Ctrl', where lower numbers indicate stronger hits in the matrix on the following page(s).

%Ctrl Calculation

$$\left(\frac{\text{test compound signal - positive control signal}}{\text{negative control signal - positive control signal}} \right) \times 100$$

test compound = compound submitted by Eurofins Cerep SA
negative control = DMSO (100%Ctrl)
positive control = control compound (0%Ctrl)

Relationship between Binding Constant Distributions (Kds) &Single Concentration Primary Screen Values

Based on screening data from thousands of profiled compounds, a proportional relationship between primary screening results and corresponding compound/target affinities may be described. Evident in the correlation graph below is a range of binding constants (Kd values) for the indicated ranges of POC values with tighter binding (higher affinity) interactions associated with lower POC values and weaker binding (lower affinity) associated with higher POC values. This distribution of binding constants is characteristic of single concentration primary screens and underscores the importance of following up observed 'hits' or apparent high affinity interactions with quantitative binding constant determinations.

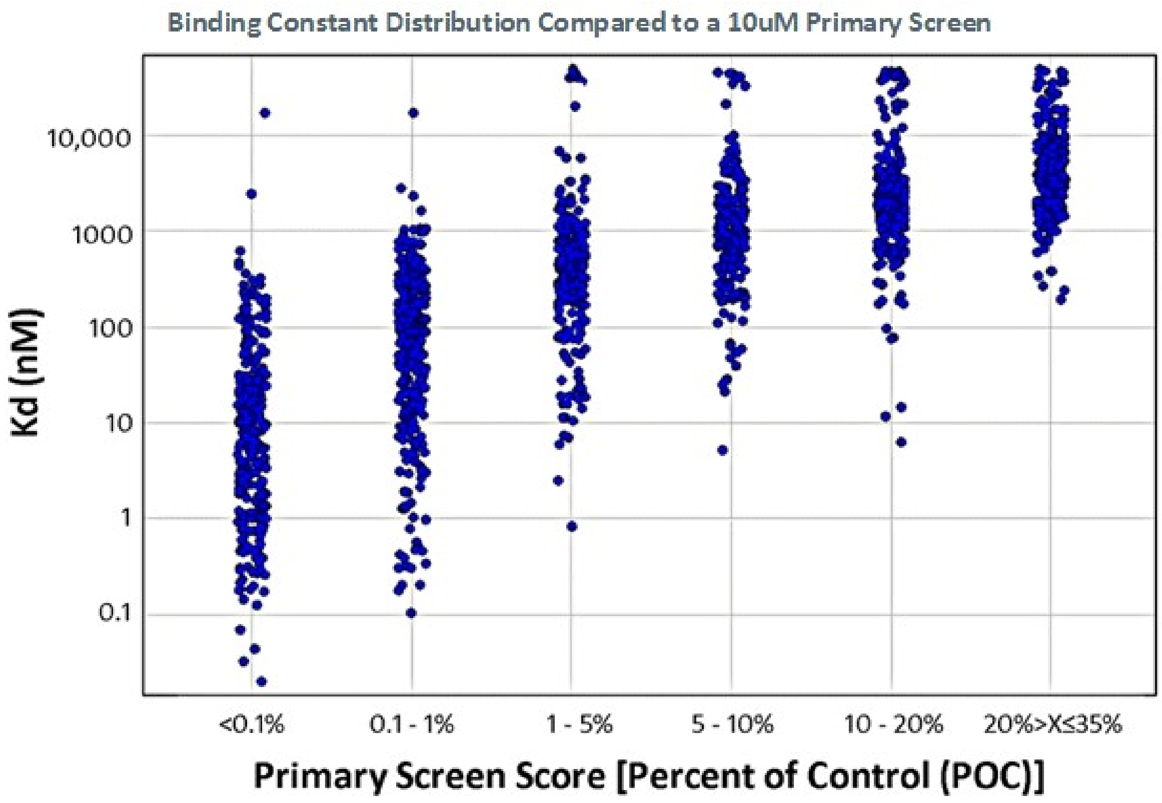

Data correlation between primary screening (10µM concentration) and binding constants (Kd values). Binding constants are correlated with primary screening results, where lower POC values are associated with low Kd values (higher affinity interactions).

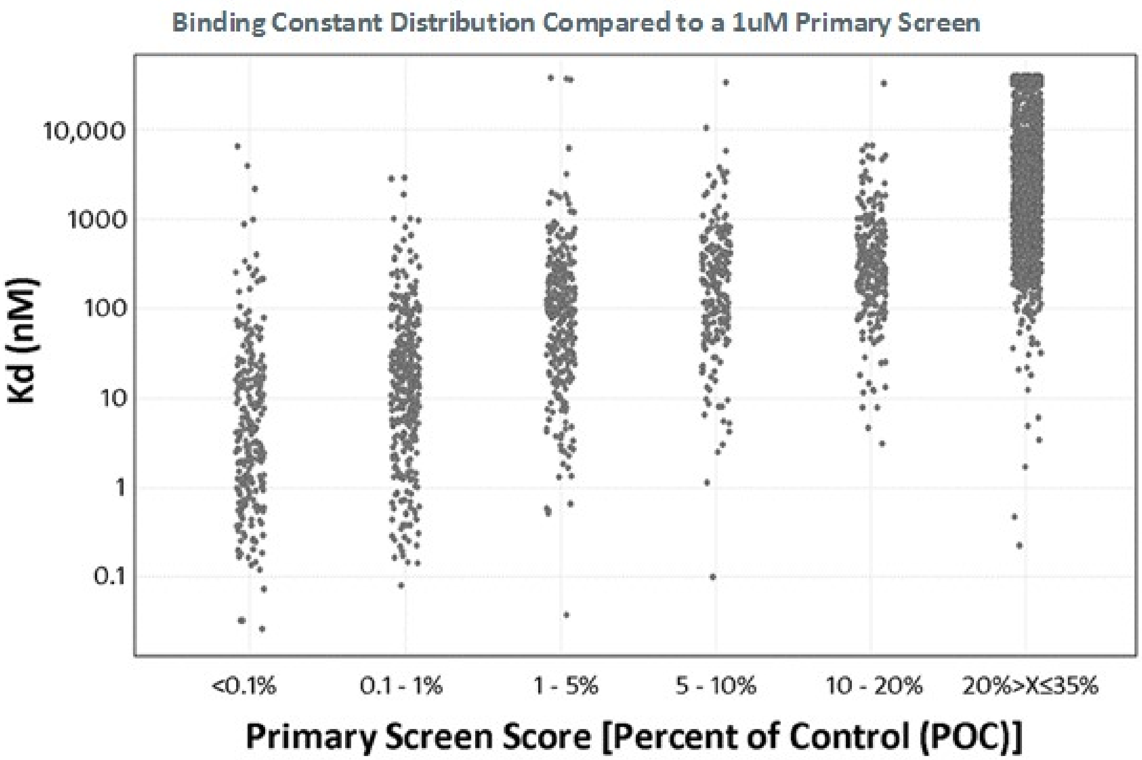

Data correlation between primary screening (1μM concentration) and binding constants (Kd values). Binding constants are correlated with primary screening results, where lower POC values are associated with low Kd values (higher affinity interactions).

Selectivity Score (S-scores)

Selectivity Score or S-score is a quantitative measure of compound selectivity. It is calculated by dividing the number of kinases that compounds bind to by the total number of distinct kinases tested, excluding mutant variants.

S = Number of hits / Number of assays

This value can be calculated using %Ctrl as a potency threshold (below) and provides a quantitative method of describing compound selectivity to facilitate comparison of different compounds.

S(35) = (number of non-mutant kinases with %Ctrl <35)/(number of non-mutant kinases tested)
S(10) = (number of non-mutant kinases with %Ctrl <10)/(number of non-mutant kinases tested)
S(1) = (number of non-mutant kinases with %Ctrl <1)/(number of non-mutant kinases tested)

Using S-Score Data to Quantitate Selectivity

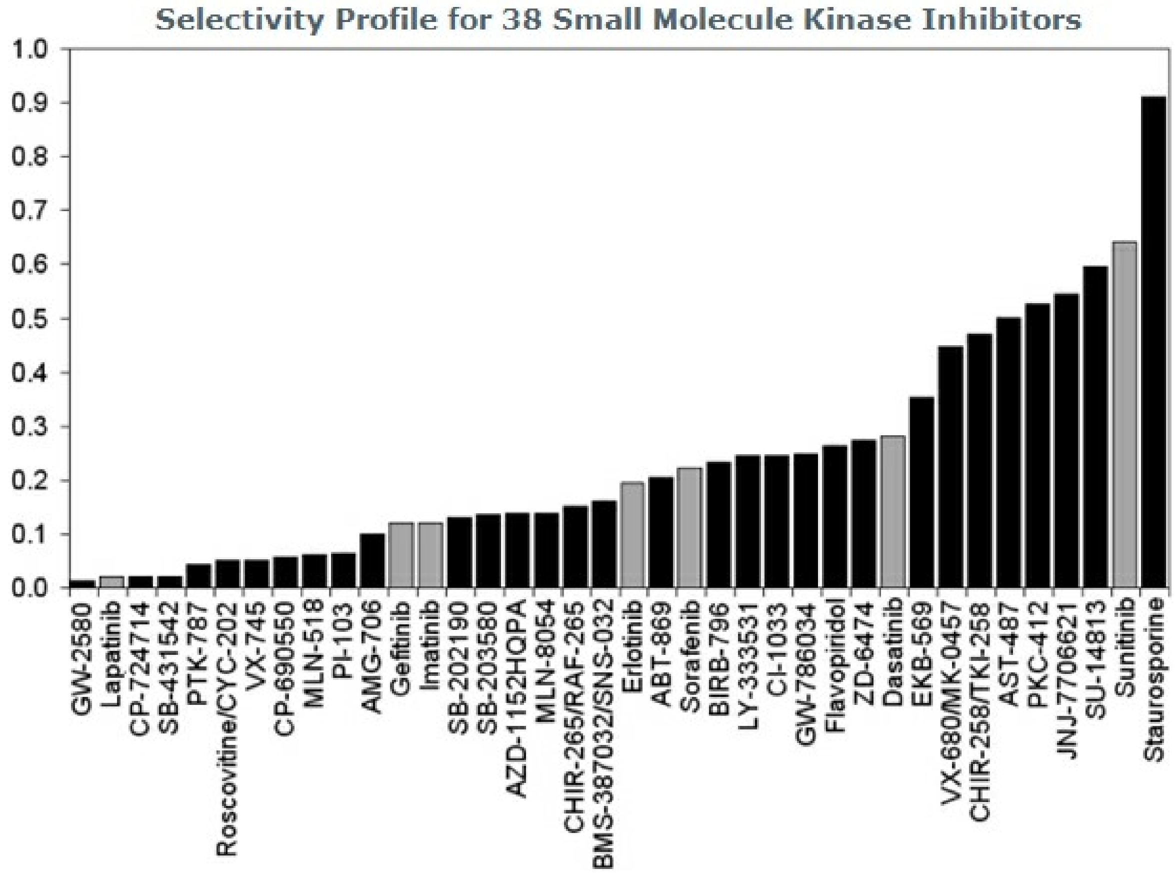

KINOMEscan's in vitro competition binding assay was used to evaluate 38 kinase inhibitors against a panel of 287 distinct human protein kinases (~55% of the predicted human protein kinome), and three lipid kinases. The compounds tested included 21 tyrosine kinase inhibitors, 15 serine-threonine kinase inhibitors, 1 lipid kinase inhibitor and staurosporine. S(35) = (number of non-mutant kinases with %Ctrl <35)/(290 kinases tested; 27 mutant variants were excluded from this analysis). Compounds approved for use in humans (as of August, 2007) are highlighted (gray bars).

References

KINOME*scan*™ and BROMO*scan*™ use the same assay technology. For a more detailed description of this assay technology, see:

• Fabian, M.A. *et al.* A small molecule-kinase interaction map for clinical kinase inhibitors. *Nat. Biotechnol.* **23**, 329-336 (2005).

To view kinase interaction maps for 38 well-known kinase inhibitors and a more detailed discussion of selectivity scores, see:

• Karaman, M.W. *et al.* A quantitative analysis of kinase inhibitor selectivity. *Nat. Biotechnol.* 26, 127-132 (2008).

Select publications are available at www.discoverx.com.

EUR184-01-p-00001 Study Results

Table 1 - Matrix of Compound Screen for EUR184-01-p-00001

Target	AB668
Gene Symbol	%Ctrl @ 2000nM
AAK1	80
ABL1(E255K)-phosphorylated	67
ABL1(F317I)-nonphosphorylated	79
ABL1(F317I)-phosphorylated	96
ABL1(F317L)-nonphosphorylated	88
ABL1(F317L)-phosphorylated	78
ABL1(H396P)-nonphosphorylated	97
ABL1(H396P)-phosphorylated	80
ABL1(M351T)-phosphorylated	86
ABL1(Q252H)-nonphosphorylated	73
ABL1(Q252H)-phosphorylated	86
ABL1(T315I)-nonphosphorylated	64
ABL1(T315I)-phosphorylated	79
ABL1(Y253F)-phosphorylated	95
ABL1-nonphosphorylated	96
ABL1-phosphorylated	82
ABL2	94
ACVR1	100
ACVR1B	75
ACVR2A	95
ACVR2B	92
ACVRL1	90
ADCK3	86
ADCK4	80
AKT1	88
AKT2	97
AKT3	92
ALK	91
ALK(C1156Y)	81
ALK(L1196M)	85
AMPK-alpha1	99
AMPK-alpha2	100
ANKK1	100
ARK5	83
ASK1	100
ASK2	75
AURKA	100
AURKB	99
AURKC	100
AXL	100
BIKE	99
BLK	100
BMPR1A	77
BMPR1B	100
BMPR2	100
BMX	85
BRAF	100
BRAF(V600E)	100

Table 1 - Assay Matrix (continued).

Target	AB668
Gene Symbol	%Ctrl @ 2000nM
BRK	72
BRSK1	78
BRSK2	79
BTK	100
BUB1	94
CAMK1	77
CAMK1B	100
CAMK1D	96
CAMK1G	80
CAMK2A	96
CAMK2B	96
CAMK2D	89
CAMK2G	78
CAMK4	100
CAMKK1	88
CAMKK2	81
CASK	97
CDC2L1	90
CDC2L2	94
CDC2L5	98
CDK11	76
CDK2	90
CDK3	100
CDK4	100
CDK4-cyclinD1	84
CDK4-cyclinD3	96
CDK5	99
CDK7	98
CDK8	81
CDK9	93
CDKL1	86
CDKL2	84
CDKL3	88
CDKL5	91
CHEK1	95
CHEK2	100
CIT	88
CLK1	67
CLK2	98
CLK3	79
CLK4	97
CSF1R	100
CSF1R-autoinhibited	85
CSK	95
CSNK1A1	96
CSNK1A1L	86
CSNK1D	89
CSNK1E	66
CSNK1G1	90

Table 1 - Assay Matrix (continued).

Target	AB668
Gene Symbol	%Ctrl @ 2000nM
CSNK1G2	75
CSNK1G3	67
CSNK2A1	0.6
CSNK2A2	19
CTK	82
DAPK1	100
DAPK2	71
DAPK3	100
DCAMKL1	71
DCAMKL2	93
DCAMKL3	91
DDR1	96
DDR2	94
DLK	83
DMPK	90
DMPK2	85
DRAK1	100
DRAK2	100
DYRK1A	99
DYRK1B	83
DYRK2	76
EGFR	92
EGFR(E746-A750del)	88
EGFR(G719C)	83
EGFR(G719S)	68
EGFR(L747-E749del, A750P)	90
EGFR(L747-S752del, P753S)	98
EGFR(L747-T751del,Sins)	86
EGFR(L858R)	96
EGFR(L858R,T790M)	83
EGFR(L861Q)	79
EGFR(S752-I759del)	80
EGFR(T790M)	89
EIF2AK1	88
EPHA1	99
EPHA2	92
EPHA3	86
EPHA4	78
EPHA5	100
EPHA6	83
EPHA7	93
EPHA8	92
EPHB1	100
EPHB2	98
EPHB3	80
EPHB4	79
EPHB6	82
ERBB2	100
ERBB3	94

Table 1 - Assay Matrix (continued).

Target	AB668
Gene Symbol	%Ctrl @ 2000nM
ERBB4	97
ERK1	75
ERK2	100
ERK3	91
ERK4	84
ERK5	88
ERK8	53
ERN1	100
FAK	91
FER	100
FES	91
FGFR1	81
FGFR2	93
FGFR3	89
FGFR3(G697C)	100
FGFR4	82
FGR	72
FLT1	85
FLT3	87
FLT3(D835H)	89
FLT3(D835V)	94
FLT3(D835Y)	79
FLT3(ITD)	93
FLT3(ITD,D835V)	77
FLT3(ITD,F691L)	96
FLT3(K663Q)	96
FLT3(N841I)	95
FLT3(R834Q)	77
FLT3-autoinhibited	100
FLT4	98
FRK	70
FYN	64
GAK	99
GCN2(Kin.Dom.2,S808G)	80
GRK1	80
GRK2	100
GRK3	89
GRK4	98
GRK7	97
GSK3A	84
GSK3B	100
HASPIN	100
HCK	96
HIPK1	100
HIPK2	100
HIPK3	98
HIPK4	87
HPK1	92
HUNK	88

Table 1 - Assay Matrix (continued).

Target	AB668
Gene Symbol	%Ctrl @ 2000nM
ICK	100
IGF1R	73
IKK-alpha	100
IKK-beta	91
IKK-epsilon	76
INSR	97
INSRR	93
IRAK1	98
IRAK3	86
IRAK4	92
ITK	93
JAK1(JH1domain-catalytic)	75
JAK1(JH2domain-pseudokinase)	93
JAK2(JH1domain-catalytic)	85
JAK3(JH1domain-catalytic)	73
JNK1	77
JNK2	92
JNK3	92
KIT	95
KIT(A829P)	78
KIT(D816H)	58
KIT(D816V)	95
KIT(L576P)	91
KIT(V559D)	98
KIT(V559D,T670I)	93
KIT(V559D,V654A)	100
KIT-autoinhibited	96
LATS1	100
LATS2	89
LCK	99
LIMK1	100
LIMK2	84
LKB1	89
LOK	100
LRRK2	65
LRRK2(G2019S)	86
LTK	94
LYN	88
LZK	100
MAK	96
MAP3K1	91
MAP3K15	51
MAP3K2	93
MAP3K3	100
MAP3K4	90
MAP4K2	100
MAP4K3	82
MAP4K4	96
MAP4K5	96

Table 1 - Assay Matrix (continued).

Target	AB668
Gene Symbol	%Ctrl @ 2000nM
MAPKAPK2	92
MAPKAPK5	95
MARK1	79
MARK2	81
MARK3	0
MARK4	87
MAST1	100
MEK1	97
MEK2	96
MEK3	100
MEK4	89
MEK5	100
MEK6	83
MELK	100
MERTK	96
MET	100
MET(M1250T)	90
MET(Y1235D)	74
MINK	100
MKK7	82
MKNK1	63
MKNK2	100
MLCK	100
MLK1	96
MLK2	73
MLK3	99
MRCKA	84
MRCKB	90
MST1	97
MST1R	97
MST2	97
MST3	100
MST4	92
MTOR	92
MUSK	92
MYLK	81
MYLK2	96
MYLK4	100
MYO3A	96
MYO3B	100
NDR1	93
NDR2	100
NEK1	73
NEK10	80
NEK11	100
NEK2	90
NEK3	71
NEK4	86
NEK5	84

Table 1 - Assay Matrix (continued).

Target	AB668
Gene Symbol	%Ctrl @ 2000nM
NEK6	87
NEK7	93
NEK9	94
NIK	72
NIM1	84
NLK	77
OSR1	81
p38-alpha	87
p38-beta	74
p38-delta	96
p38-gamma	74
PAK1	99
PAK2	81
PAK3	95
PAK4	95
PAK6	90
PAK7	85
PCTK1	94
PCTK2	96
PCTK3	91
PDGFRA	86
PDGFRB	100
PDPK1	98
PFCDPK1(P.falciparum)	81
PFPK5(P.falciparum)	100
PFTAIRE2	88
PFTK1	84
PHKG1	76
PHKG2	88
PIK3C2B	100
PIK3C2G	100
PIK3CA	82
PIK3CA(C420R)	100
PIK3CA(E542K)	99
PIK3CA(E545A)	100
PIK3CA(E545K)	88
PIK3CA(H1047L)	98
PIK3CA(H1047Y)	100
PIK3CA(I800L)	98
PIK3CA(M1043I)	100
PIK3CA(Q546K)	100
PIK3CB	90
PIK3CD	91
PIK3CG	90
PIK4CB	75
PIKFYVE	1.7
PIM1	91
PIM2	84
PIM3	85

Table 1 - Assay Matrix (continued).

Target	AB668
Gene Symbol	%Ctrl @ 2000nM
PIP5K1A	85
PIP5K1C	87
PIP5K2B	100
PIP5K2C	81
PKAC-alpha	84
PKAC-beta	78
PKMYT1	70
PKN1	91
PKN2	85
PKNB(M.tuberculosis)	100
PLK1	100
PLK2	87
PLK3	93
PLK4	94
PRKCD	91
PRKCE	98
PRKCH	92
PRKCI	85
PRKCQ	87
PRKD1	100
PRKD2	88
PRKD3	100
PRKG1	85
PRKG2	97
PRKR	75
PRKX	69
PRP4	95
PYK2	95
QSK	85
RAF1	100
RET	87
RET(M918T)	94
RET(V804L)	93
RET(V804M)	76
RIOK1	80
RIOK2	73
RIOK3	97
RIPK1	96
RIPK2	83
RIPK4	100
RIPK5	95
ROCK1	88
ROCK2	83
ROS1	89
RPS6KA4(Kin.Dom.1-N-terminal)	88
RPS6KA4(Kin.Dom.2-C-terminal)	97
RPS6KA5(Kin.Dom.1-N-terminal)	95
RPS6KA5(Kin.Dom.2-C-terminal)	43
RSK1(Kin.Dom.1-N-terminal)	100

Table 1 - Assay Matrix (continued).

Target	AB668
Gene Symbol	%Ctrl @ 2000nM
RSK1(Kin.Dom.2-C-terminal)	100
RSK2(Kin.Dom.1-N-terminal)	61
RSK2(Kin.Dom.2-C-terminal)	81
RSK3(Kin.Dom.1-N-terminal)	93
RSK3(Kin.Dom.2-C-terminal)	85
RSK4(Kin.Dom.1-N-terminal)	88
RSK4(Kin.Dom.2-C-terminal)	94
S6K1	89
SBK1	88
SGK	100
SgK110	71
SGK2	98
SGK3	99
SIK	97
SIK2	100
SLK	100
SNARK	94
SNRK	100
SRC	92
SRMS	93
SRPK1	88
SRPK2	98
SRPK3	100
STK16	82
STK33	67
STK35	89
STK36	100
STK39	90
SYK	100
TAK1	84
TAOK1	100
TAOK2	100
TAOK3	100
TBK1	87
TEC	95
TESK1	95
TGFBR1	84
TGFBR2	90
TIE1	89
TIE2	83
TLK1	100
TLK2	88
TNIK	88
TNK1	96
TNK2	100
TNNI3K	96
TRKA	96
TRKB	87
TRKC	100

Table 1 - Assay Matrix (continued).

Target	AB668
Gene Symbol	%Ctrl @ 2000nM
TRPM6	81
TSSK1B	91
TSSK3	98
TTK	85
TXK	92
TYK2(JH1domain-catalytic)	85
TYK2(JH2domain-pseudokinase)	100
TYRO3	100
ULK1	77
ULK2	100
ULK3	82
VEGFR2	98
VPS34	100
VRK2	78
WEE1	96
WEE2	95
WNK1	100
WNK2	87
WNK3	98
WNK4	96
YANK1	78
YANK2	76
YANK3	92
YES	100
YSK1	82
YSK4	100
ZAK	100
ZAP70	76

%Ctrl Legend

0≤x<.1	.1≤x<1	1≤x<10	10≤x<35	x≥35

S-score Results

Table 2 - S-score Table for EUR184-01-p-00001

Compound Name	Selectivity Score Type	Number of Hits	Number of Non-Mutant Kinases	Screening Concentration (nM)	Selectivity Score
AB668	S(35)	4	403	2000	0.01
AB668	S(10)	3	403	2000	0.007
AB668	S(1)	2	403	2000	0.005

TREE*spot*™ Interaction Maps - Now Includes Mutant, Lipid, Atypical & Pathogen Kinase Dendrograms

As part of our ongoing effort to provide customers with the best possible data analysis tools, KINOME*scan*™ has developed an enhanced rendering of the human kinase dendrogram and allows, for the first time ever, to fully visualize compound interactions across our industry leading kinase panel, including clinically and biochemically relevant mutants, lipid, atypical, and pathogen kinases, plus a growing panel of activation-state specific assays.

TREE*spot*™ is an artistic representation of the human kinome phylogenetic tree based on extensive published research. We welcome your comments and feedback on this new visualization image. Please contact us at info@discoverx.com to tell us what you think.

Key Changes

- More uniform format and presentation
- Kinase groups more clearly delineated
- Updated nomenclature for kinases

TREE*spot*™ is a proprietary data visualization software tool developed by KINOME*scan*. *Mutant and lipid kinases are not represented.* Kinases found to bind are marked with red circles, where larger circles indicate higher-affinity binding. Visualize data online and create your own high resolution TREE*spot*™ interaction maps with our easy-to-use compound profile visualization tool. Instructions and login credentials provided below.

Login: treespot.discoverx.com -- **Username:** treespot! -- **Password:** guest037
Instructions: treespot.discoverx.com/Help/TreeSpotHelpBasic.htm

Table 3 - TREE*spot*™ Interaction Maps for EUR184-01-p-00001

AB668 @ 2000nM

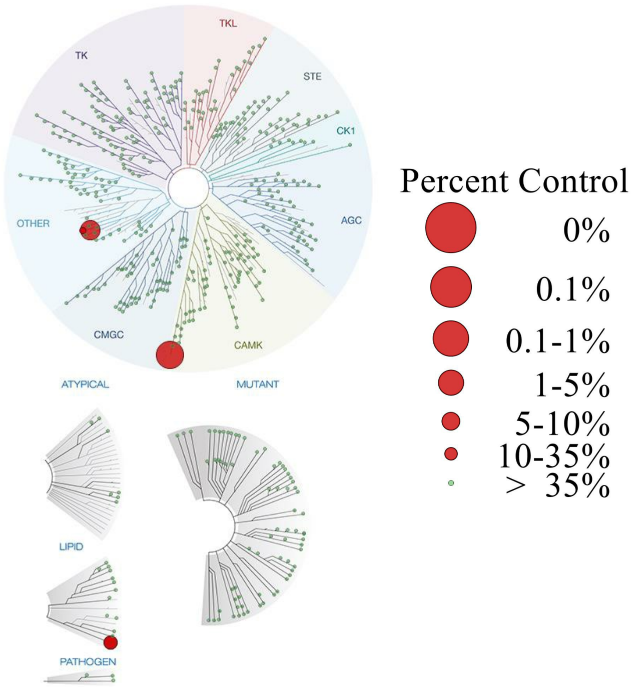

Available Follow-up Screening Services

LeadHunter™ Discovery Services offers a suite of investigative tools that enable detailed biochemical characterization of the interaction between inhibitors and their targets. The thermodynamic, kinetic, and structural information provided by these tools enables a detailed comparison of inhibitors from common or distinct lead series and facilitates the interpretation of data from downstream cellular and *in vivo* pharmacology models. These services are now available for both kinases and for bromodomain-containing proteins.

---

kdELECT Obtain quantitative binding affinities for compound-kinase interactions

Kd**ELECT™** - a powerful follow up service to quantify binding affinity of compound-kinase interactions identified in primary (single concentration) screens. Inhibitor binding constants (Kd values) are calculated from duplicate 11-point dose-response curves under optimized conditions that generate true thermodynamic Kd values which facilitate direct comparison of inhibitor affinity across kinases. Learn more >>

Now Available!

PathHunter® cell-based compound screening & profiling services

PathHunter inCell assays and screening services are a powerful follow up solution to KINOME*scan*™ *in vitro* biochemical studies for obtaining the maximum level of information about inhibitor function, potency and selectivity in a more physiological context.

Technology Overview

KINOME*scan*™ is the industry's most comprehensive high-throughput system for screening compounds against large numbers of human kinases. Developed by DiscoverX, KINOME*scan*™ employs proprietary active-site dependent competition binding assays to determine how compounds bind to both intended and unintended kinases. In addition to helping keep discovery programs on track, KINOME*scan*™ can opportunistically identify unanticipated interactions that can expand the therapeutic utility of compounds or serve as advanced starting points for new programs.

How KINOME*scan*™ Works

KINOME*scan*™ is based on a competition binding assay that quantitatively measures the ability of a compound to compete with an immobilized, active-site directed ligand. The assay is performed by combining three components: DNA-tagged kinase; immobilized ligand; and a test compound. The ability of the test compound to compete with the immobilized ligand is measured via quantitative PCR of the DNA tag.

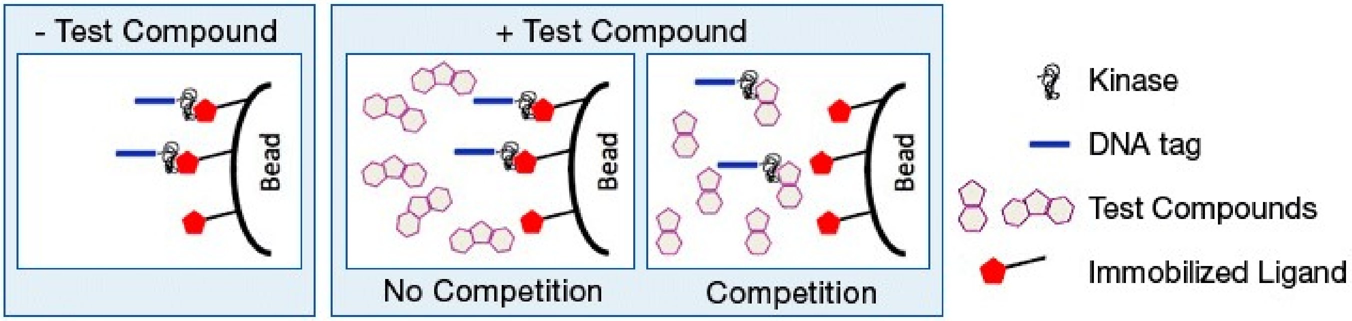

Protocol Description

Kinase assays. For most assays, kinase-tagged T7 phage strains were prepared in an *E. coli* host derived from the BL21 strain. *E. coli* were grown to log-phase and infected with T7 phage and incubated with shaking at 32°C until lysis. The lysates were centrifuged and filtered to remove cell debris. The remaining kinases were produced in HEK-293 cells and subsequently tagged with DNA for qPCR detection. Streptavidin-coated magnetic beads were treated with biotinylated small molecule ligands for 30 minutes at room temperature to generate affinity resins for kinase assays. The liganded beads were blocked with excess biotin and washed with blocking buffer (SeaBlock (Pierce), 1% BSA, 0.05% Tween 20, 1 mM DTT) to remove unbound ligand and to reduce non-specific binding. Binding reactions were assembled by combining kinases, liganded affinity beads, and test compounds in 1x binding buffer (20% SeaBlock, 0.17x PBS, 0.05% Tween 20, 6 mM DTT). Test compounds were prepared as 111X stocks in 100% DMSO. Kds were determined using an 11-point 3-fold compound dilution series with three DMSO control points. All compounds for Kd measurements are distributed by acoustic transfer (non-contact dispensing) in 100% DMSO. The compounds were then diluted directly into the assays such that the final concentration of DMSO was 0.9%. All reactions performed in polypropylene 384-well plate. Each was a final volume of 0.02 ml. The assay plates were incubated at room temperature with shaking for 1 hour and the affinity beads were washed with wash buffer (1x PBS, 0.05% Tween 20). The beads were then re-suspended in elution buffer (1x PBS, 0.05% Tween 20, 0.5 μM non-biotinylated affinity ligand) and incubated at room temperature with shaking for 30 minutes. The kinase concentration in the eluates was measured by qPCR.

Compound Handling

An 11-point 3-fold serial dilution of each test compound was prepared in 100% DMSO at 100x final test concentration and subsequently diluted to 1x in the assay (final DMSO concentration = 1%). Most Kds were determined using a compound top concentration = 30,000 nM. If the initial Kd determined was < 0.5 nM (the lowest concentration tested), the measurement was repeated with a serial dilution starting at a lower top concentration. A Kd value reported as 40,000 nM indicates that the Kd was determined to be >30,000 nM.

Binding Constants (Kds)

Binding constants (Kds) were calculated with a standard dose-response curve using the Hill equation:

$$\text{Response} = \text{Background} + \frac{\text{Signal - Background}}{1 + (\text{Kd}^{\text{Hill Slope}} / \text{Dose}^{\text{Hill Slope}})}$$

The Hill Slope was set to -1.

Curves were fitted using a non-linear least square fit with the Levenberg-Marquardt algorithm.

TREE*spot*™ Compound Profile Visualization Tool

TREE*spot*™ is a proprietary data visualization software tool developed by KINOME*scan*™. Visualize data online and create your own high resolution TREE*spot*™ interaction maps with our easy-to-use compound profile visualization tool. TREE*spot*™ is provided as a complimentary service to our clients. To access TREE*spot*™, please follow these directions:

Login: treespot.discoverx.com -- **Username:** treespot! -- **Password:** guest037
Instructions: treespot.discoverx.com/Help/TreeSpotHelpBasic.htm

References

KINOME*scan*™ and BROMO*scan*™ use the same assay technology. For a more detailed description of this assay technology, see:

- Fabian, M.A. *et al*. A small molecule-kinase interaction map for clinical kinase inhibitors. *Nat. Biotechnol.* **23**, 329-336 (2005).

To view kinase interactions for 38 well-known kinase inhibitors, see:

- Karaman, M.W. *et al*. A quantitative analysis of kinase inhibitor selectivity. *Nat. Biotechnol.* **26**, 127-132 (2008).

For examples on how KINOME*scan* can opportunistically identify unanticipated therapeutically-beneficial interactions, see:

- Carter, T.A. *et al*. Inhibition of drug-resistant mutants of ABL, KIT, and EGF receptor kinases. *Proc. Natl. Acad. Sci.* USA. **102**, 11011-11016 (2005)

For more information on the Hill equation and the Levenberg-Marquardt algorithm, see:

- Hill, A. V. The possible effects of the aggregation of the molecules of hemoglobin on its dissociation curves. *J. Physiol. (Lond.)*. **40**, iv-vii (1910).
- Levenberg, K. A method for the solution of certain non-linear problems in least squares. *Q. Appl. Math.* **2**, 164-168 (1944).

Select publications are available at www.discoverx.com.

EUR191-01-s-00001 Study Results

Table 1 - Matrix of Kds for EUR191-01-s-00001.

Target	AB668
Gene Symbol	Kd (nM)
CSNK2A1	86
MARK3	>10000
PIKFYVE	>10000

Kd Legend

x<100nM	100nM≤x<1uM	x≥1uM	No Binding	Not Requested

EUR191-01-s-00001 Curve Images

Table 2 - Curve Images for EUR191-01-s-00001. The amount of kinase measured by qPCR (Signal; y-axis) is plotted against the corresponding compound concentration in nM in log10 scale (x-axis). Data points marked with an "x" were not used for Kd determination.

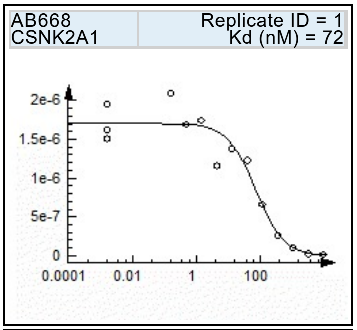

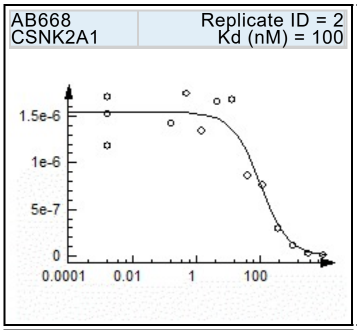

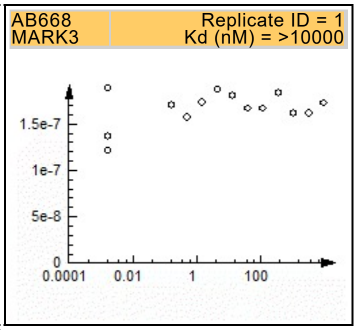

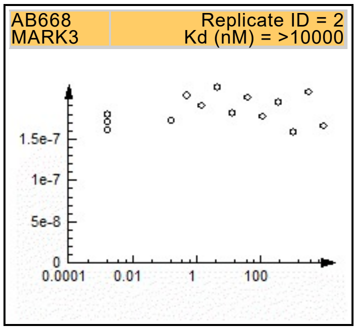

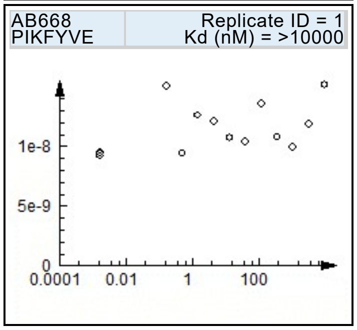

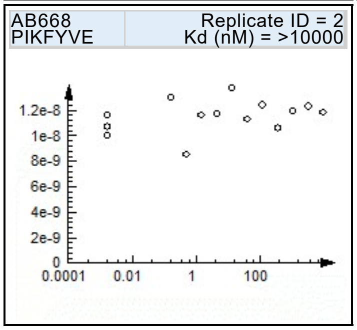

Supplement: Data S1. Kinase scan for AB668, performed using KINOMEscan™ screening platform, related to Figure 1 [file mmc2.pdf]
